# Efficacy of a breastfeeding support education program for nurses and midwives: a randomized controlled trial

**DOI:** 10.1186/s13006-022-00532-2

**Published:** 2022-12-22

**Authors:** Izumi Sato, Masumi Imura, Yohei Kawasaki

**Affiliations:** 1 Japanese Red Cross College of Nursing, Saitama, Japan; 2 Maternal Nursing, Japanese Red Cross College of Nursing, 8-7-19 Kamiochiai Saitama-shi, Chuo-ku, Saitama, 338-0001 Japan; 3 Global Health Care and Midwifery Graduate School of Nursing, Japanese Red Cross College of Nursing, Tokyo, Japan

**Keywords:** Breastfeeding, Educational program, Late preterm infants, Randomized controlled trial

## Abstract

**Background:**

Nutritional support influences the growth and development of late preterm infants (LPIs) and their long-term health status. However, healthy LPIs have a shorter hospital stay and may not receive adequate care after discharge. In this study, we developed and evaluated the effectiveness of an educational program for nurses and midwives to enable them to support breastfeeding of healthy LPIs.

**Methods:**

A randomized controlled trial was conducted in Japan from July 2018 to April 2019. The participant pool consisted of nurses and midwives working at midwiferies and obstetric centers in Tokyo, Japan. A total of 395 candidates were recruited for participation across 79 facilities. The final participants were assigned to two groups: the breastfeeding support for LPIs program (BSLPI group; *n* = 36) or the non-technical skills program (NTS group; *n* = 33). The measures included the Self-Efficacy of Breastfeeding Support scale (SBS), the Social Skills in Nursing Interactions with Mothers (SS) scale, and the Knowledge and Skills Necessary for Breastfeeding Support for LPIs test (K-S). Scores for each measure were collected before, after, and one-month after the intervention. Repeated-measures ANOVA was used to identify differences (main effects) according to program (BSLPI and NTS) and time (before, immediately after, and one month after intervention).

**Results:**

All 69 participants attended the program. Main effects of the program were observed only for K-S scores (*F*[1,58] = 78.57, *p* = 0.01). No significant differences were found for SBS (*F*[1,58] = 0.63, *p* = 0.43) or SS scores (*F*[1,58] = 1.51, *p* = 0.23).

**Conclusions:**

Participation in the BSLPI was related to improved breastfeeding support knowledge and skills but was not related to improvements in nurses’ self-efficacy or social skills.

**Trial registration:**

Registered 12 December 2018, https://center6.umin.ac.jp/cgi-open-bin/ctr/ctr_view.cgi?recptno=R000040145 (UMIN: UMIN000035227).

**Supplementary Information:**

The online version contains supplementary material available at 10.1186/s13006-022-00532-2.

## Background

Infants born between 34 weeks and 0 days and 36 weeks and 6 days of gestation are called late preterm infants (LPIs). There is an urgent worldwide need for innovative solutions to reduce preterm birth rates to protect preterm infants, including LPIs, from various risks [1]. Nutritional support in infancy influences future health status, and breastmilk intake is expected to improve the body composition of LPIs to an optimal state, positively influence future health maintenance [2], and potentially optimize gut flora [3].

Breastfeeding is promoted for all neonates [4], and LPIs are no exception. Though breastmilk is the optimal form of nutrition, adhering to the Ten Steps to Successful Breastfeeding [4] alone may not help mothers cope with the challenges that arise after hospital discharge [5, 6]. A survey on LPIs who did not require medical intervention and were capable of oral feeding demonstrated that they were significantly less likely to be breastfed than term infants [7–10]. Compared with term infants, LPIs may need to wait longer for the mother to secrete breastmilk. In fact, regarding LPI feeding methods in hospitals, 10% dextrose and infant formula are reportedly administered from the early postnatal period for LPIs capable of oral feeding [11]. Although further investigations are required, prevention of LPI hypoglycemia, poor weight gain, and ensuring that mothers and their babies can be discharged from the hospital as scheduled are some factors that might explain these practices. Jensen reported that overfeeding LPIs with supplementary sources, such as dextrose and formula, can potentially affect latching and suckling and lower breastmilk supply [12]. Further, mothers of LPIs who used additional methods for feeding during the hospital stay and the first week at home were more likely to use a bottle to feed their infant at one month compared with mothers of term infants [7]. Alternatively, an abundant supply of breastmilk in the first week of hospitalization or at home is a factor that enables LPIs to be exclusively fed breastmilk in the first month of life [7].

Against this background, we believe that direct breastfeeding should, whenever possible, be the basis for LPIs and full-term infants. Further, nurses and midwives should be trained to provide scientifically based support for a smooth transition from feeding aids to direct breastfeeding.

In a study that focused on mothers of breastfeeding LPIs, the mothers were devoted to breastfeeding from the postnatal period but experienced stress because of factors such as LPIs not waking up at feeding times [13, 14]; ineffective suckling [13, 14]; and hospital policies and unsatisfactory interactions with their physicians, nurses, and midwives [8, 13, 14]. Therefore, nurses and midwives who provide breastfeeding support for LPIs should be highly skilled in maintaining good relationships with mothers.

In Japan, healthy LPIs do not require special medical intervention even after birth despite having immature physical functions. Therefore, in the absence of abnormalities, LPIs will not be treated by a neonatologist or pediatrician and can be discharged from the maternity facilities. Mothers generally stay in the hospital for one week and are discharged together with healthy LPIs. However, LPI mothers still need continuous post-discharge support because of issues related to breastfeeding. Furthermore, out-of-pocket medical expenses and hospital visits burden LPI mothers. These factors prevent the enforcement of a specific frequency and timing for consultations. However, for healthy, maternity-ward-managed LPIs, evidence-backed support is essential from immediately after birth to the establishment of breastfeeding.

A previous study demonstrated the benefits of educational interventions in improving knowledge and attitudes among medical staff who provided care for LPIs [15]. Another study reported that when nurses, midwives, and physicians watched a video that demonstrated the necessary skills for breastfeeding support, their knowledge, acquisition of skills, and self-efficacy improved [16]. Existing educational programs for nurses and midwives who provide breastfeeding support for LPIs focus on general management, covering only fundamental topics [15]. Moreover, they are limited to techniques and do not include post-discharge breastfeeding support [16]. There are staff education programs for the systemic management of postnatal LPIs and breastfeeding support for preterm infants managed in the Neonatal Intensive Care Unit (NICU) [15, 17]. These programs have not been designed for healthy LPIs who are discharged early with their mothers. Knowledge, techniques, and communication skills are necessary for nurses and midwives to provide effective breastfeeding support and build good relationships with mothers of LPIs. Therefore, updates and improvements to the currently available programs are warranted.

This study aimed to administer and evaluate the effectiveness of an educational program designed to increase nurses’ and midwives’ self-efficacy in providing breastfeeding support and improve their social skills for maintaining good relationships with mothers. Further, nurses and midwives were expected to gain the knowledge and skills necessary for providing ongoing breastfeeding support to mothers with LPIs in the months after discharge by incorporating simulations and lectures into the program.

## Methods

### Design

**Fig. 1:**
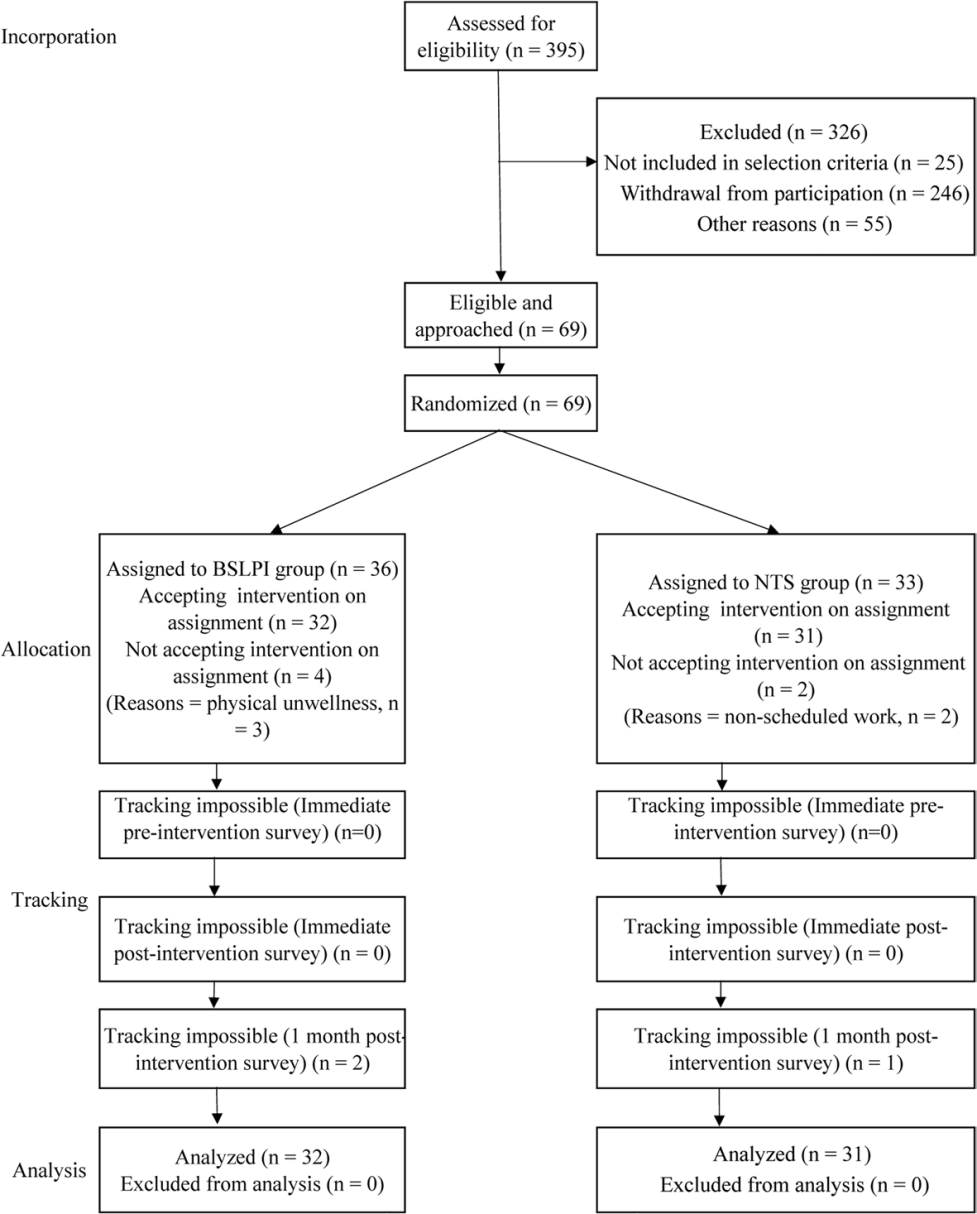
Participant selection flowchart. BSLPI: Breastfeeding support for late preterm infants; NTS: Non-technical skills

A two-group parallel randomized controlled trial was conducted (Fig. 1), and participants were assigned to one of two groups: the breastfeeding support for LPIs (BSLPI) program or the non-technical skills (NTS) program. The effects were assessed after intervention and at one-month follow-up and compared with pre-intervention assessments. We recruited participants and conducted the intervention and assessments between July 2018 and April 2019. This study was conducted according to the latest CONSORT 2010 guidelines [18] for reporting randomized parallel-group trials.

### Sample

The researchers requested cooperation from the facility managers of 160 maternity facilities in the Tokyo metropolitan area. Upon obtaining agreement for research cooperation, the facility director allowed maternity-ward managers to disseminate informative documents related to research participation to nurses and midwives managing LPIs in the wards and display posters to recruit research participants. Nurses and midwives volunteered to participate in the research by accessing the dedicated application website or by applying to participate.

The inclusion criteria were as follows: having 1) nursing or midwifery certification and working in hospitals, clinics, or midwifery centers that handle deliveries in the Tokyo metropolitan area, 2) two or more years of clinical experience in midwifery or obstetric nursing, and 3) experience in providing breastfeeding support for LPIs. The exclusion criteria were as follows: 1) not being engaged in deliveries and thus not involved in breastfeeding support for LPIs starting in the early postnatal period; and 2) being in managerial positions, such as head nurses, and not directly involved in breastfeeding support for LPIs.

### Randomization

A computerized number generator was used to achieve stratified block randomization [19], and the participants were automatically assigned to one of the two groups and four blocks according to this randomization table. Participants were assigned a symbol according to their order of registration. Each group was assigned an equal number of nurses and midwives. The researcher wrote the participant’s symbol according to the instructions in the allocation table. Tables and rosters were stored in a locked cabinet and were not accessed until randomization was completed. After randomization, participants were informed about the respective program that they were assigned to in writing.

### Blinding

Blinding of participants, researchers and research collaborators were conducted. Participants were not informed about the group they were assigned to, whether the intervention or the control group. The researchers assigned participants to the two groups automatically according to the order of an assignment table to avoid selection bias.

The data were collected by research collaborators, with the researchers conducting the analysis. The specific procedures were as follows. Measures were collected and aggregated by the research collaborators in charge of data collection and processing after the intervention. We analyzed the aggregate data in which the participants’ information was encrypted. Additionally, a statistician approved the analysis procedure to ensure objectivity and maintain integrity of the data. After tabulation, no further adjustments were made to the data, and the analysis proceeded according to the research plan.

### Sample size calculation

The was used to calculate the sample size. No randomized controlled trial has examined the effect of an educational program intervention on nurses providing breastfeeding support for LPIs. Therefore, we used the SBS score obtained from our pilot study (Additional File 1); the SBS score of the BSLPI before and after intervention was 47.5 (SD = 10.0) and 53.4 (SD = 7.6), respectively.

Statistical power analysis software G*Power [20] was used to calculate the sample size. For effect size, a Cohen’s *d* of 0.66 was calculated using historical data from the pilot study. Although this value corresponds to a large effect size, since small- to medium-effect sizes are generally recommended, a medium-effect size of 0.25 was stipulated [21]. The required sample size was 54 for an effect size = 0.25, α = 0.05, and power = 0.80, as calculated by a repeated-measures analysis of variance (ANOVA; inter-group and time factors). Assuming a 40% dropout rate, we aimed to recruit 76 participants (38 per group).

### Intervention

The intervention was implemented in two groups using two programs. The BSLPI program is a participatory educational program of lectures and exercises to improve the knowledge, skills, and perceptions necessary for breastfeeding support of LPIs. The NTS program is a non-participatory program (i.e., participants listen to the content of the lectures but do not take an active part in them) of lectures aimed at improving knowledge related to non-technical skills, such as teamwork for nurses and midwives. The BSLPI group received information on breastfeeding support for LPIs, and the NTS group did not receive breastfeeding information. The contents of the programs are shown in Table 1. The two programs contained different content and implementation methods (Additional Files 2, 3 and 4). They were delivered faithfully in accordance with the prepared schedule and scenario. The researcher was the facilitator.

**Table 1:** Implementation method and contents of each group program

Breastfeeding support education program for LPIs	Non-technical skills program
**Lecture and group work**: 1. Physical characteristics and sucking problems of LPIs 2. Characteristics and lactation problems of mothers with LPIs 3. Points and basis necessary for breastfeeding support for LPI mothers, from mid-pregnancy to post-discharge: How to do the first feeding; How to use pump; Skin-to-skin contact	**Lecture**: What are non-technical skills?
**Simulation**: Responding to LPIs during weight loss	**Lecture**: Non-technical skills in the medical field
**Social skills training**: Working with mothers with LPIs who are reluctant to breastfeed	**Lecture**: Use of non-technical skills in medical practice

*LPIs* Late preterm infants

Participants of the BSLPI group were mailed a document in advance with pertinent information, including the meeting time and venue. The participants gathered in the conference room for lectures and participatory discussions at the scheduled time. The intervention time was five hours; the program venue was a meeting room that allowed for active learning, and the seating arrangement was designed as an island. The venue was a comfortable space where participants could move freely and deepen productive discussions. The participants were given the freedom to speak freely and assured of their anonymity. They were also assured of the non-disclosure of information that could identify the facilities that participated in the program. The educational program was based on the most recent literature on breastfeeding support for LPIs [22]. Specialists in maternity nursing, pediatrics, and breastfeeding support were also consulted to increase content validity.

The participants of the NTS group were mailed a document in advance containing the details of the NTS (e.g., meeting time, venue). The research participants gathered in the conference room at the scheduled time. They introduced themselves, listened to lectures, watched videos, worked individually on given tasks, and presented their thoughts and opinions. The lecture format included only participation in the class and did not consider interaction with other participants.

The NTS education encompassed non-technical skills that could complement technical know-how [23]. Information on breastfeeding was not provided. Instead, crew resource management programs, developed to address team and leadership aspects of piloting modern airplanes [24], were utilized. Such skills and techniques play an important role in medicine and nursing [25–27].

The teaching materials used in the program comprised slides (Additional File 3), videos, and booklets that described the lecture’s content. The intervention lasted five hours, and the program location was a conference room with designated seats in a classroom seating arrangement.

To date, breastfeeding support interventions for newborns, including LPIs, include an integrative review of 13 studies comprising five randomized controlled trials and three quasi-experimental studies. The review showed that breastfeeding interventions (e.g., early mother‒infant contact, kangaroo mother care, cup feeding) effectively prolong breastfeeding duration and full breastfeeding in LPIs [28]. However, some results included the researcher’s interventions. Therefore, we trained nurses to ensure that breastfeeding interventions for LPIs could be implemented by someone other than the researcher.

In past reports, we found two educational programs that provided the knowledge and skills necessary for breastfeeding support through a before-and-after group comparison study [15, 16]. Although some of the educational content was specific to LPIs, the information was related to the knowledge and skills required to manage clinical problems and were not specific to breastfeeding [15]. Further, some were specific to breastfeeding but not to LPIs [16]. The methods of delivering educational content were video viewing [16], online learning [16], and face-to-face learning [15].

The novelty of this study is that the educational program was designed for nurses and midwives who provided breastfeeding support to the mothers of LPIs. The content targeted mothers of LPIs and focused on breastfeeding support. Further, the communication skills essential for breastfeeding support interventions for mothers were acquired through lectures and exercises.

### Assessment

The efficacy of the developed educational program was found to be sound according to measures of nurses and midwives’ SBS, SS, and knowledge and skills necessary for breastfeeding support for LPIs.

#### Self-efficacy related to breastfeeding support

The SBS scale was developed by Toyama et al. (https://www.scirp.org/journal/PaperInformation.aspx?PaperID=40906) on the basis of responses from 729 public health nurses with 5‒15 years of experience [29]. The scale indicates the quality and quantity of potential services, with self-efficacy related to breastfeeding support as a predictor of breastfeeding support behaviors; this 14-item tool was deemed reliable (Cronbach’s α = 0.89). Responses are provided on a five-point scale (1 = *not confident at all*; 5 = *extremely confident*). The total score ranges from 14 to 70, with high scores indicating a high sense of self-efficacy in breastfeeding support. The developer’s permission was obtained to use the scale.

#### Social skills in nursing interactions with mothers

Social skills encompass the verbal and non-verbal interpersonal behaviors used to respond appropriately and effectively in interpersonal situations and the cognitive processes that enable such expression [30]. Fusa et al. developed the Social Skills for Nursing as Novice Nurse’s scale (http://www-nurs.iwate-pu.ac.jp/journal/) [31]. For this study, one item, “consultation with patients about life after discharge,” was deleted because it was deemed inadequate as a social skill. Study subjects included nurses and midwives who were not involved during hospitalization but began their involvement after discharge. Thus, it was anticipated that some study subjects might be confused when responding to this item, and it was eliminated to ensure accurate responses.

The reliability and validity of the scale after deleting this item were assessed. The factor structure of the responses of 18 participants was tested to confirm that there were no major changes from the original scale to test the scale’s validity. Furthermore, internal consistency was confirmed (Cronbach’s α = 0.85). The developer’s permission was obtained to use the revised version. All processes were carried out with a maternity nursing specialist, and the new scale was used to test social skills in nursing interactions with mothers (SS). The participants assessed these 24 items on a four-point scale (1 = *never used*; 4 = *always used*), and the total possible scores ranged from 24 to 96, with high scores indicating the use of more verbal and non-verbal social skills.

#### Knowledge and skills necessary for breastfeeding support for LPIs

We created a 20-item questionnaire evaluating knowledge and skills (K-S) to assess participants’ knowledge of breastfeeding support for LPIs. It consisted of two items on basic knowledge about LPIs, 14 items on knowledge and skills related to breastfeeding support for their mothers, and four items on knowledge of systemic management of LPIs (Additional File 5). The test was performed at regular intervals to evaluate the consistency of the measurement items. The same items were measured separately for the first and second times, thus confirming their consistency (Additional File 6). The items scored five points for each correct answer, with the total score ranging from 0 to 100. The higher the scores, the better their knowledge and skills.

### Data collection

Participants were recruited from July 2018 to March 2019, and data were collected between July 2018 and April 2019. The pre- and post-intervention data were collected at the convention center, and the one-month follow-up data were collected by mail. Immediately before the interventions, information on age, years of clinical experience, years of working in maternity wards, breastfeeding experience, nurse or midwife status, hospital facility type, and SBS, SS, and K-S test scores were obtained. SBS, SS, and K-S test scores were completed directly after the interventions and at one-month follow-up.

### Data analysis

The descriptive statistics for each variable (degree, range, mean, standard deviation) were calculated. The Shapiro‒Wilk test was used to assess the normality of the scores for the scales. The baseline scores for the SBS, SS, and K-S tests were t-tested to evaluate the similarity between the two groups.

For the SBS, SS, and K-S, repeated-measures ANOVA [32] was conducted to determine whether significant differences existed in the mean scores between the two programs (BSLPI and NTS) and whether significant differences existed in mean scores due to time (immediately before, immediately after, and one month after intervention). Missing values were treated without using any substitution or imputation method.

First, tests of sphericity (Mauchly’s sphericity test and Box’s test for equality of covariance matrixes) [33] were conducted. Sphericity was assumed for *p*-values > 0.05, while the variance results of the Greenhouse‒Geisser ε correction were evaluated for *p*-values < 0.05.

The interactions (Program×Time) between the variables were analyzed to identify where they significantly changed the mean scores. For significant interactions (*p* < 0.05), multiple comparisons were performed. Each program factor was divided according to the time factor, and multiple comparisons were conducted. Equality of variance testing (Levene’s test) was performed, and the Tukey method was adopted for *p*-values ≥ 0.05. Additionally, the Games‒Howell test was adopted for *p*-values < 0.05, following which the Bonferroni correction was performed. A corrected *p*-value < 0.05 was considered significant. A Bonferroni correction was performed to prevent a Type 1 error in the multiple comparisons test.

Multiple comparisons between levels were performed for any factor for which the main effect *p*-value was < 0.05 and the interaction *p*-value was > 0.005. The repeated-measures factor was significant. Equality of variance testing (Levene’s test) was performed first, and the Tukey method was adopted for *p*-values > 0.05. Further, the Games‒Howell test was adopted for *p*-values < 0.05, following which the Bonferroni correction was performed.

To clarify the difference in scores based on the study participants’ characteristics, repeated-measures ANOVA was performed to confirm significant differences in the SS, SBS, and K-S test scores in groups with less than five years and five or more years of clinical experience. *P* < 0.05 was considered statistically significant, and SPSS Statistics 28.0 (IBM Corp., Armonk, NY, USA) was used for the analyses.

## Results

Nine sessions each were held for the BSLPI and NTS programs, totaling 18 sessions. The number of participants (mean) for each session was 5.3 (SD = 3.5) for BSLPI and 3.4 (SD = 2.6) for NTS. A total of 395 candidates were recruited for participation across 79 facilities. Of these, 326 individuals were removed, including 25 who did not meet the inclusion criteria, 246 who left the study, and 55 who were excluded for other reasons. Ultimately, 69 individuals agreed to participate in the study and were included in the analysis.

Of the 69 nurses and midwives who applied to participate in the study, 36 and 33 were randomly assigned to the intervention and control groups, respectively. Three participants in the intervention group did not undergo the intervention because of poor health and one because of an unexpected change in work schedule. Two participants in the control group could not undergo the intervention because of an unexpected change in their work schedule. The number of participants in the intervention and control groups who could be studied before intervention, after intervention, and at one-month follow-up were 32 (89.9%) and 31 (94.0%), 32 (83.4%) and 31 (91.0%), and 30 (83.4%) and 30 (91.0%), respectively. Three participants who underwent the intervention but could not be followed up (4.8%) were treated as missing data without supplementation. The data from immediately after the intervention were imputed such that 63 participants were ultimately included in the analysis (follow-up rate 87.0%; Fig. 1).

The similarity between the BSLPI and NTS groups was confirmed (Tables 2, 3, and 4). The ages and years of experience in midwifery or obstetric nursing of participants in the BSLPI and NTS groups were 37.8 years (*SD* = 11.5) and 38.8 years (*SD* = 8.1) and 10.7 years (*SD* = 9.8) and 10.4 years (*SD* = 6.6), respectively. The participants were a mix of skilled professionals with many years of clinical experience and participants with fewer years of experience. Approximately half of each group had breastfed. Hospitals accounted for the largest number of affiliated facilities, comprising half of each group. Regarding the highest educational attainment, 53.1% (*n* = 17) of participants in the BSLPI group and 38.7% (*n* = 12) in the NTS group had bachelor’s degrees or higher.

**Table 2:** Two-group comparison by age and years of experience

Item	BSLPI (*n* = 32)		NTS (*n* = 31)	
	*Mean*	*SD*	*Mean*	*SD*
**Age**	37.8	11.5	38.8	8.1
**Years of experience**	12.2	10.1	12.9	7.4
**Years of midwifery or obstetric nursing experience**	10.7	9.8	10.4	6.6

*SD* Standard deviation, *BSLPI* Breastfeeding support for late preterm infants, *NTS* Non-technical skill

**Table 3:** Inter-group comparison of participants’ characteristics

Item	BSLPI (*n* = 32)		NTS (*n* = 31)	
	*n*	%	*n*	%
**Experience of breastfeeding themselves(Y/N)**				
** Y**	15	46.9	20	64.5
** N**	17	53.1	11	35.5
**Nurses, midwives** ^a^ **(No.)**				
** Midwives**	30	93.8	28	90.3
** Nurses**	2	6.3	2	6.5
**Facility status**				
** Hospital**	20	62.5	18	58.1
** Clinic**	4	12.5	6	19.4
** Midwife center**	8	25.0	7	22.6
**Educational background**				
** University**	17	53.1	12	38.7
** Professional educational institution**	15	46.9	19	61.3

*BSLPI* Breastfeeding support for late preterm infants, *NTS* Non-technical skills

^a^ In Japan, midwives are certified professionals

**Table 4:** Inter-group baseline comparison by each evaluative index

	BSLPI (*n* = 32)		NTS (*n* = 31)		*t*	*df*	*P*
	*Mean*	*SD*	*Mean*	*SD*			
**SBS scale scores**	47.8	10.3	50.5	7.6	1.2	61	0.25
≤ **5 years**	41.8	6.7	48.9	4.6	2.6	19	0.02
** 6 or more years**	51.9	10.4	51.0	8.4	0.3	40	0.77
**SS scale scores**	74.2	11.0	74.6	8.9	0.2	61	0.22
** ≤ 5 years**	71.9	13.8	72.3	11.6	0.6	19	0.96
** ≥ 6 years**	75.8	9.2	75.5	7.9	1.4	40	0.89
** K-S test scores**	44.8	12.0	45.7	12.0	0.3	61	0.93
** ≤ 5 years**	42.6	11.1	49.6	7.5	0.9	19	0.36
** ≥ 6 years**	46.3	12.7	45.2	13.3	0.3	40	0.79

*BSLPI* Breastfeeding support for late preterm infants, *NTS* Non-technical skills, *LPIs* Late preterm infants, *SBS* Self-efficacy of breastfeeding support, *SS* Social skills in nursing interactions with mothers, *K-S* Knowledge and skills necessary for breastfeeding support for LPIs

Further, there were similarities in baseline scores for the SBS (BSLPI = 47.8, SD = 10.3; NTS = 50.5, SD = 7.6), SS (BSLPI = 74.2, SD = 11.0; NTS = 74.6, SD = 8.9), and K-S (BSLPI = 44.8, SD = 12.0; NTS = 45.7, SD = 12.0) scales. In the subgroup with five or fewer years of experience in the maternity ward, the SBS score was higher in the NTS group than in the BSLPI group (Table 4).

### Repeated-measures ANOVA for each measure

The SBS scale, SS scale, and K-S test scores were analyzed using repeated-measures ANOVA to determine whether significant differences existed in scores (main effect) for each program (BSLPI and NTS) and time (before, immediately after, and one month after intervention; Tables 5, 6, 7, 8 and 9). Results indicated a main effect for program difference for K-S scores only (*F*[1,58] = 78.57, *p* = 0.01), with no significant differences observed for SBS (*F*[1,58] = 0.63, *p* = 0.43) or SS scores (*F*[1,58] = 1.51, *p* = 0.23). Main effects for time were observed for SBS (*F*[1.74,101.00] = 24.19, *p* = 0.01), SS (*F*[1.79,103.16] = 15.59, *p* = 0.01) and K-S scores (*F*[1.60,92.61] = 10805.42, *p <* 0.01). Program⋅Time interactions were observed for all measures (SBS: *F*[1.74,101.00] = 8.94, *p* = 0.01; SS: *F*[1.78,103.16] = 8.32, *p* = 0.01; K-S: *F*[1.60,92.61] = 77.46, *p* = 0.295).

**Table 5:** Mean scores for both programs over time

Between-group: Program			BSLPI						NTS			
Within-group: Time	Pre		Post		1 M		Pre		Post		1 M	
	*Mean/SE*		*Mean/SE*		*Mean/SE*		*Mean/SE*		*Mean/SE*		*Mean/SE*	
**SBS**	47.6	1.7	55.6	1.4	57.4	1.5	50.7	1.7	51.8	1.4	53.8	1.5
** SBS ≤ 5 y**	41.6	1.8	52.8	1.7	56.3	2.1	49.4	2.3	50.9	2.2	52.0	2.8
**SS**	73.7	1.8	80.2	1.8	81.8	1.7	75.0	8.9	75.7	1.8	76.5	1.7
** SS ≤ 5 y**	71.7	3.8	80.2	3.2	83.8	3.0	73.1	4.2	75.0	9.7	74.6	3.9
**K-S**	45.7	2.2	84.8	1.9	79.7	2.2	45.5	2.2	48.8	1.9	52.0	2.2
** K-S ≤ 5 y**	42.5	3.0	84.6	2.4	77.5	3.1	46.4	4.0	49.3	3.2	48.6	4.0
** K-S ≥ 6y**	47.8	2.9	85.0	2.7	81.1	3.0	48.7	2.4	48.7	12.5	53.0	2.7

*SE* Standard error, *BSLPI* Breastfeeding support for late preterm infants, *NTS* Non-technical skills, *LPIs* Late preterm infants, *SBS* Self-efficacy of breastfeeding support, *SS* Social skills in nursing interactions with mothers, *K-S* Knowledge and skills necessary for breastfeeding support for LPIs, *5y* 5 or less years of maternity-ward experience, *6y* 6 or more years of maternity-ward experience

**Table 6:** Analysis of variance assessing the SBS scale

			*SS*	*df*	*MS*	*F*	*P*
**SBS Scale Scores ** ^a^	**Within-group factor**	**Time** **Time×Program**	1318.300 462.700	1.741 1.741	757.080 265.722	24.189 8.940	0.01 0.01
	***MSe*** ^c^		3161.000	100.995	31.299		
	**Between-group factor**	**Program**	101.250	1	101.250	0.632	0.43
	***MSe***		9291.700	58	160.202		
**≤ 5 years** ^b^	**Within-group factor**	**Time** **Time×Program**	705.434 365.364	2 2	352.717 182.682	25.227 13.066	0.01 0.01
	***MSe***		475.373	34	13.982		
	**Between-group factor**	**Program**	3.863	1	3.863	0.039	0.85
	***MSe***		1678.032	17	98.708		
**≥ 6 years** ^b^	**Within-group factor**	**Time** **Time×Program**	554.208 119.021	2 2	277.104 59.510	8.832 1.897	0.01 0.16
	***MSe***		2447.142	78	31.374		
	**Between-group factor**	**Program**	305.005	1	305.005	1.755	0.19
	***MSe***		6776.295	39	173.751		

*SBS* Self-efficacy of breastfeeding support, *SS* Social skills in nursing interactions with mothers, *K-S* Knowledge and skills necessary for breastfeeding support for LPIs, *5 or fewer years* 5 or fewer years of maternity-ward experience, *6 or more years* 6 or more years of maternity-ward experience, *SS* Sum of Squares, *df* degrees of freedom, *MS* Mean squares, *F* ANOVA test statistic, *p* *p*-value

^a^ Analysis of variance with Greenhouse‒Geisser

^b^ Analysis of variance with Mauchly’s sphericity test

^c^ Mean squares

**Table 7:** Analysis of variance assessing the SS scale

			*SS*	*df*	*MS*	*F*	*p*
**SS Scale Score** ^a^	**Within-group factor**	**Time** **Time×Program**	749.678 400.011	1.779 1.779	421.516 224.911	15.587 8.317	0.01 0.01
	***MSe***		2789.64	103.155	27.043		
	**Between-group factor**	**Program**	366.939	1	366.936	1.505	0.23
	***MSe***		14137.789	58	243.755		
**≤ 5 years** ^a^	**Within-group factor**	**Time** **Time×Program**	398.749 300.222	1.361 1.361	293.008 220.609	5.746 4.326	0.02 0.04
	***MSe***		1179.78	23.135	50.995		
	**Between-group factor**	**Program**	310.860	1	310.860	0.93	0.35
	***MSe***		5674.58	17	333.795		
**≥ 6 years** ^b^	**Within-group factor**	**Time** **Time×Program**	336.654 99.678	2 2	168.327 49.839	8.466 2.507	0.01 0.09
	***MSe***		1550.826	78	19.882		
	**Between-group factor**	**Program**	123.524	1	123.524	0.597	0.44
	***MSe***		8071.826	39	206.970		

*SBS* Self-efficacy of breastfeeding support, *SS* Social skills in nursing interactions with mothers, *K-S* Knowledge and skills necessary for breastfeeding support for LPIs, *5 or fewer years* 5 or fewer years of maternity-ward experience, *6 or more years* 6 or more years of maternity-ward experience, *SS* Sum of Squares, *df* degrees of freedom, *MS* Mean squares, *F* ANOVA test statistic, *p* *p*-value

^a^ Analysis of variance with assumption of sphericity

^b^ Analysis of variance with Greenhouse‒Geisser

**Table 8:** Analysis of variance assessing the K-S scale

			*SS*	*df*	*MS*	*F*	*P*
**K-S Test Score** ^b^	**Within-group factor**	**Time** **Time×Program**	17252.500 10548.611	1.597 1.597	10805.420 6606.705	126.682 77.456	0.01 0.01
	***MSe***		7898.889	92.606	85.296		
	**Between-group factor**	**Program**	20373.472	1	20373.472	78.566	0.01
	***MSe***		15040.278	58	259.315		
**≤ 5 years** ^a^	**Within-group factor**	**Time** **Time×Program**	5099.008 3918.306	2 2	2549.504 1959.153	39.088 30.037	0.01 0.01
	***MSe***		2217.659	34	65.225		
	**Between-group factor**	**Program**	5358.025	1	5358.025	33.145	0.01
	***MSe***		1748.115	17	161.654		
**≥ 6 years** ^b^	**Within-group factor**	**Time** **Time×Program**	11278.430 6250.787	1.448 1.448	7791.126 4318.036	79.650 44.144	0.01 0.01
	***MSe***		5522.383	56.456	97.817		
	**Between-group factor**	**Program**	15078.828	1	15078.828	49.260	0.01
	***MSe***		11938.245	39	306.109		

*SBS* Self-efficacy of breastfeeding support, *SS* Social skills in nursing interactions with mothers, *K-S* Knowledge and skills necessary for breastfeeding support for LPIs, *5 or fewer years* 5 or fewer years of maternity-ward experience, *6 or more years* 6 or more years of maternity-ward experience, *SS* Sum of Squares, *df* degrees of freedom, *MS* Mean squares, *F* ANOVA test statistic, *p p-*value

^a^ Analysis of variance with Assumption of sphericity

^b^ Analysis of variance with Greenhouse‒Geisser

**Table 9:** Repeated-measures analysis of variance: unpaired/paired)

	Main effects		Interaction	Multiple comparisons (Time/Program)
	Time	Program	Time×Program	Corrected *p*
**SBS**	*		*	**Time** BSLPI: Pre < Post (*p* = 0.01); BSLPI: Pre < 1 M (*p* = 0.01)
** SBS ≤ 5 y**	*		*	**Time** BSLPI: Pre < Post (*p* = 0.01); BSLPI: Pre < 1 M (*p* = 0.01)
** SBS ≥ 6y**	*			**Time** BSLPI: Pre < Post (*p* = 0.01); BSLPI: Pre < 1 M (*p* = 0.01)
**SS**	*		*	**Time** BSLPI: Pre < Post (*p* = 0.01); BSLPI: Pre < 1 M (*p* = 0.01)
** SS ≤ 5 y**	*		*	**Time** BSLPI: Pre < Post (*p* = 0.01); BSLPI: Pre < 1 M (*p* = 0.01)
** SS ≥ 6y**	*			**Time** BSLPI: Pre < Post (*p* = 0.04); BSLPI: Pre < 1 M (*p* = 0.01)
**K-S**	*	*	*	**Time** BSLPI: Pre < Post (*p* = 0.01); BSLPI: Pre < 1 M (*p* = 0.01) **Program** Post: BSLPI > NTS (*p* = 0.01); Post 1 M: BSLPI > NTS (*p* = 0.01)
** K-S ≤ 5 y**	*	*	*	**Time** BSLPI: Pre < Post (*p* = 0.01); BSLPI: Pre < 1 M (*p* = 0.01) **Program** Post: BSLPI > NTS (*p* = 0.01); Post 1 M: BSLPI > NTS (*p* = 0.01)
** K-S ≥ 6y**	*	*	*	**Time** BSLPI: Pre < Post (*p* = 0.01); BSLPI: Pre < 1 M (*p* = 0.01) **Program** Post: BSLPI > NTS (*p* = 0.01); Post 1 M: BSLPI > NTS (*p* = 0.01)

Multiple comparisons of time factors (Bonferroni correction)

Multiple comparisons of program factors (Bonferroni correction)

*SBS* Self-efficacy of breastfeeding support, *SS* Social skills in nursing interactions with mothers, *K-S* Knowledge and skills necessary for breastfeeding support for LPIs, *SBS* High scores indicate a high sense of self-efficacy in breastfeeding support, *SS* High scores indicate the use of more verbal and non-verbal social skills, *K-S* High scores indicate better knowledge and skills, *5 years* Five or fewer years of maternity-ward experience, *6 or more years* Six or more years of maternity-ward experience. BSLPI group (*n* = 32): 13 < 5 years, 19 ≥ 5 years. NTS group (*n* = 31): 8 < 5 years, 23 ≥ 5 years; *p* *p-*value

### Multiple comparisons for each measure

Multiple comparisons and Bonferroni corrections were performed to determine where significant differences between the program and time factors were observed for the interactions (Tables 5 and 9).

In terms of program factors, BSLPI scores were significantly higher compared with NTS only on the K-S test scores (Pre: BSLPI: M = 84.80, SD = 1.90 > NTS: M = 48.80, SD = 1.90, *p* = 0.01; 1 M: BSLPI: M = 79.70, SD = 2.20 > NTS: M = 52.00, SD = 2.20, *p* = 0.01).

For the time factors, the BSLPI group had significantly higher scores after intervention compared with before intervention (SBS: Pre: M = 47.60, SD = 1.70 < Post: Pre: M = 55.60, SD = 1.40, *p* = 0.01; Pre: M = 47.60, SD = 1.70 < 1 M: M = 57.40, SD = 1.50, *p* = 0.01; SS: Pre: M = 73.70, SD = 1.80 < Post: Pre: M = 80.20, SD = 1.80, *p* = 0.01; Pre: M = 73.70, SD = 1.80 < 1 M: M = 81.80, SD = 1.70, *p* = 0.01; K-S: Pre: M = 45.70, SD = 2.20 < Post: M = 84.80, SD = 1.90, *p* = 0.01; Pre: M = 45.70, SD = 2.20 < 1 M: M = 79.70, SD = 2.20, *p* = 0.01).

### Maternity-ward experience subgroup differences

Tables 5, 6, 7, 8 and 9 provide a detailed analysis results of participants divided according to years of clinical experience, either less than 5 years or more than 6 years.

## Discussion

This two-group parallel randomized controlled trial examined a specialized education program designed to improve breastfeeding support for LPIs. An effect of the BSLPI program was demonstrated only for the K-S test scores (Table 9). The current findings indicated similar results for nurses with ≤ 5 years’ and ≥ 6 years’ experience working in a maternity ward (Table 9). The BSLPI program was shown to be effective in improving the knowledge and skills of nurses providing breastfeeding support for LPIs, regardless of their maternity-ward experience duration. When the scores of the two groups were compared over time, the BSLPI group demonstrated significantly higher scores across all three measures, with similar patterns of scores being observed regardless of maternity-ward experience (Table 9).

### Self-efficacy related to breastfeeding support

We believe that the relationship between the SBS score and the BSLPI program is as follows (Table 9). Although there is scientific evidence indicating the benefits of support for breastfeeding LPIs [22], in clinical practice, first-time breastfeeding, early mother-infant contact, and kangaroo mother-infant care are not as actively supported for LPIs as they are for full-term infants. To supplement this support, participants in the BSLPI group considered and shared feasible methods to ensure that each facility could easily provide these care practices without any delay or obstacles. In addition, we provided lectures and exercises (Table 1) in the BSLPI program, including components of self-efficacy for breastfeeding support. As a result, the SBS score increased after intervention and at one-month follow-up.

### Social skills in nursing interactions with mothers

The SS score of the participants increased both after intervention and at one-month follow-up. The SS score illustrates how often a social skill is used in real-life situations. Therefore, by acquiring social skills through training, the frequency of social skills used after intervention and one month later is higher than that before intervention. Accordingly, we contend that social skill training is a well-established intervention that results in a stable increase in social skill scores.

### Knowledge and skills necessary for breastfeeding support for LPIs

Regarding the relationship between K-S test scores and the BSLPI program, studies assessing the effectiveness of educational interventions in medical facilities that provide breastfeeding support have reported that such interventions allow medical staff to improve their knowledge [34, 35]. However, we could not identify any studies wherein the interventions were specific to breastfeeding support for LPIs and that assessed the benefits of such a program. Therefore, we created a test of the knowledge and skills necessary for breastfeeding support based on guidelines developed by experts [22, 36]. The higher scores are attributed to the fact that the content of expert guidelines was summarized and explained, making it easier for participants to comprehend. Breastfeeding support for LPIs requires strategic planning and execution [22]. Therefore, we created a simple chart depicting the types of care required at various stages based on references such as the Ten Steps to Successful Breastfeeding [4], the Academy of Breastfeeding Medicine Clinical Protocol #10 [22], and the Baby-friendly Hospital Initiative for Neonatal Wards (Neo-BFHI) Core document [37]. Participants used this chart during group work and took it home after the program, allowing them to apply their acquired knowledge and skills in their workplaces.

### Strengths

The BSLPI was developed by seriously considering the issues faced in the breastfeeding of LPIs, as raised by the nurse/midwife and the mother/LPI, and thus from two important perspectives. These perspectives comprise new ideas in staff education for breastfeeding support. It further clarifies the LPIs’ and mothers’ real support needs.

The program we developed included the clarification of issues at each facility and facilitated sharing among participants; it helped in acquiring knowledge to address the relevant issues, used exercises to integrate knowledge and skills, and implemented the approach of pretend play among the participants. This program is expected to be used to improve the skills of nurses and midwives involved in breastfeeding support for LPIs.

Regarding the clinical application at an organizational level, we hope that nurses and midwives involved in providing breastfeeding support for LPIs will create systems and workflows detailing who will provide which intervention and at what stage. This will elucidate the specific functions to be performed and how to do so. Furthermore, we believe that the quality of breastfeeding support for LPIs can be improved throughout the organization by using, evaluating, and revising the program material.

Nurses who provide breastfeeding support for LPIs face different challenges owing to the LPIs’ physical immaturity. Considering diverse challenges, nurses should share a common understanding of the essential knowledge and skills necessary for providing breastfeeding support for LPIs and provide this support. We carefully selected the most basic knowledge and skills necessary for breastfeeding support for LPIs and created an educational program. We believe that this educational program, when used in practice, will help ensure the quality of LPIs’ breastfeeding support.

### Limitations

This study has some limitations (Additional Files 7 and 8):

- The sample size was relatively small. Consequently, we could not perform sufficient analysis with adjustments or sufficiently validate the program effects.
- Undeniably, the sample selection method may have created a selection bias for those who volunteered of their own free will. It is possible that the participants were highly interested in breastfeeding support and wanted to acquire knowledge and skills related to LPIs. There could have been a volunteer bias caused by the intrusion of the participants’ will. In the future, further refinement of the sample selection method is necessary.
- The researcher conducted the intervention directly in the two programs as a lecturer. Therefore, there may have been bias due to the Rosenthal effect [38] in each group. The researcher could have expected the BSLPI group to improve their knowledge and skills necessary for breastfeeding support, and this expectation may have been expressed verbally and physically. Participants may have strived to improve their knowledge and skills in breastfeeding support to meet the researcher’s expectations. Alternatively, participants in the NTS group may not have strived to improve their knowledge and skills in breastfeeding support because their expectations were different from those in the BSLPI group. To credibly test the effectiveness of an educational program in future studies, the researcher must avoid directly intervening with the participants.
- The SBS scale [29] has not been used before and needs to be further refined using the results of this study as a tool to appropriately measure breastfeeding self-efficacy. The SBS scale should be used in different regions and for different targets in the future to ensure its reliability and validity.
- The program was implemented in a central city in Japan. Considering an appropriate implementation method while ensuring the quality of the program content will increase the likelihood that incumbent nurses and midwives working in various regions have access to the educational program. A wide range of age groups participated in this study, some with minimal and others with extensive maternity-ward experience. They were all interested in breastfeeding support for LPI. Therefore, nurses and midwives who are motivated to learn about breastfeeding should be able to access the program. Regional disparities in breastfeeding support for LPIs should be eliminated and a system that allows LPIs to receive high-quality support regardless of residence created. For this purpose, facilitators should be trained to implement the BSLPI program.
- The clinical trial was registered after the commencement of this study. Such studies should be implemented only after informing the relative parties to ensure transparency [18].
- Although participants were not informed about the group they were assigned to (whether the intervention or the control group), the intervention was conducted by the researchers; thus, the method of blinding may have been insufficient [18].
- The evaluation of knowledge, skills, and attitudes used in this study is considered a valid method of verifying the effectiveness of the educational programs. However, significant improvements in participants’ scores were found for only one measure. Therefore, further refinement may be necessary for using this measure in clinical practice.

## Conclusion

A BSLPI intervention effect was demonstrated only for K-S test scores. However, a time-based comparison of the two groups’ scores indicated significantly higher scores for the BSLPI group across all three measures versus the NPI group. Results for participants with ≤ 5 years of experience working in a maternity ward were similar for both interventions; however, for nurses with ≥ 6 years of experience working in a maternity ward, only the BSLPI group had significantly higher scores on the K-S test.

## Supplementary Information

**Additional file 1.** BSLPI pilot study.

**Additional file 2.** Details of the two programs: content and implementation method.

**Additional file 3.** Lecture slides.

**Additional file 4.** Breastfeeding support chart for LPIs.

**Additional file 5.** Knowledge and skills necessary for breastfeeding support for LPIs (K-S test).

**Additional file 6.** Scale development: knowledge and skills necessary for breastfeeding support for LPIs.

**Additional file 7.** Cochrane review risk of bias checklist.

**Additional file 8.** CONSORT 2010 checklist.

## Data Availability

Not applicable.

## Abbreviations

LPI: Late preterm infant
BSLPI: Breastfeeding support for late preterm infants
NTS: Non-technical skills
SBS: Self-efficacy of breastfeeding support
SS: Social skills in nursing interactions with mothers
K-S: Knowledge and skills necessary for breastfeeding support for LPIs
