# Supplementary material for: Efficacy of a breastfeeding support education program for nurses and midwives: a randomized controlled trial
Source: Int Breastfeed J. 2022 Dec 22;17:92. doi: 10.1186/s13006-022-00532-2 (PMC9773528; doi:10.1186/s13006-022-00532-2)
Supplement: Supplementary file 1 — Additional file 1. BSLPI pilot study. [file 13006_2022_532_MOESM1_ESM.pdf]

BSLPI pilot study

The pilot study was conducted to check the feasibility of the two types of programs. The evaluation periods were pre-intervention, post-intervention, and at one-month follow-up. The analysis included 11 participants each in the BSLPI and NTS groups. The analysis was conducted using the Friedman test in each group. The results showed that in the BSLPI group, there were significant changes in the self-efficacy of breastfeeding support (SBS) scale scores ($\chi^2 = 14.5$, $df = 2$, $p = 0.01$) and knowledge and skills (K-S) test scores ($\chi^2 = 16.7$, $df = 2$, $p = 0.01$). There were no significant changes in social skills in nursing interactions with mothers' scores ($\chi^2 = 7.9$, $df = 2$, $p = 0.02$).

In the NTS group, however, there was no significant change in the SBS ($\chi^2 = 4.8$, $df = 2$, $p = 0.10$), SS ($\chi^2 = 4.2$, $df = 2$, $p = 0.16$), or K-S ($\chi^2 = 14.5$, $df = 2$, $p = 0.9$) scores.

Therefore, we supposed that the BSLPI could be implemented by slightly revising the intervention method for SS, which did not reveal significant differences.
