# Supplementary material for: Efficacy of a breastfeeding support education program for nurses and midwives: a randomized controlled trial
Source: Int Breastfeed J. 2022 Dec 22;17:92. doi: 10.1186/s13006-022-00532-2 (PMC9773528; doi:10.1186/s13006-022-00532-2)
Supplement: Supplementary file 2 — Additional file 2. Details of the two programs: content and implementation method. [file 13006_2022_532_MOESM2_ESM.pdf]

Details of the two programs: content and implementation method	
Breastfeeding support education program for LPIs (BSLPI)	Non-technical skills program (NTS)
Orientation (20 min)	Orientation (15 min)
1. Physical characteristics and suckling problems of LPIs (33 min)<br>Lecture: Introduction, definition of LPIs, etc.<br>Group work: Our image of LPIs<br>Characteristics and lactation problems of mothers with LPIs (24 min)<br>2. Characteristics of mothers who breastfeed their LPIs<br>Lecture: Characteristics of mothers of LPIs<br>Group work: How does a mother of an LPI feel?<br>3. Points and basis necessary for breastfeeding support for LPI mothers, from mid-pregnancy to post-discharge (52 min)<br>Lecture: Support to make direct breastfeeding top priority<br>Group work: How to perform the first feeding; how to use a pump; skin-to-skin contact	1. Differences between non-technical skills and technical skills (15 min)<br>Lecture: What are non-technical skills?<br>Watch the video: What are non-technical skills?<br>2. Thinking to find and solve problems (88 min)<br>Lecture：What is problem solving?<br>Watch the video: Causes and solutions to problems
4. Let's try breastfeeding support in practice	3. How to solve problems in an

(74 min)<br>Simulation: Case — "Responding to LPIs during weight loss"<br>Take turns playing the role of the nurse, mother, and observer	organization (80 min)<br>Personal work: Your own analysis<br>Watch the video: The power of organization
5. How to communicate with mothers with LPIs in the early stages of breastfeeding (55 min)<br>Social skills training: Case — "Working with mothers with LPIs who are reluctant to breastfeed"<br>Take turns playing the role of the nurse, mother, and observer.	4. How to motivate yourself (40 min)<br>Exchange of views: All participants
Closing (10 min)	Closing (10 min)

BSLPI

Participants were divided into small groups, where they introduced themselves and discussed the implementation status and issues surrounding breastfeeding support at each facility based on the lectures. A simulation was conducted to integrate the input knowledge and skills into an output. The simulation was conducted in small groups, with study participants taking turns playing the roles of mothers, nurses, or midwives. The case studies were based on situations that nurses and midwives often encounter in breastfeeding support for LPIs (e.g., excessive weight loss and decreased feeding ability). The nurses used the knowledge and skills acquired in the lecture to assess the breastfeeding situation and consider the necessary care. The mothers played the role of

mothers of LPIs. A debriefing was conducted.

Finally, social skills training was conducted using cases of mothers breastfeeding LPIs. Most of this program was based on intra-group collaborations with other participants.

The teaching materials comprised slides containing the contents of the lecture, booklet, breastfeeding support chart for LPIs (additional file), breast and newborn baby models for breastfeeding practice, and a wrap (stretchable cloth) that facilitates movement while maintaining skin-to-skin contact.

NTS

Participants did not interact with other participants and attended lectures. The content of the lecture was a definition and overview of non-technical skills and their actual use in the medical field.
