# Supplementary material for: Efficacy of a breastfeeding support education program for nurses and midwives: a randomized controlled trial
Source: Int Breastfeed J. 2022 Dec 22;17:92. doi: 10.1186/s13006-022-00532-2 (PMC9773528; doi:10.1186/s13006-022-00532-2)
Supplement: Supplementary file 3 — Additional file 3. Lecture slides. [file 13006_2022_532_MOESM3_ESM.pdf]

Preterm babies
22 weeks 0 days to 36 weeks 6 days

Full-term baby
37 weeks 0 days to 41 weeks 6 days

34 weeks 0 days to 36 weeks 6 days
Late Preterm Infant (LPI)

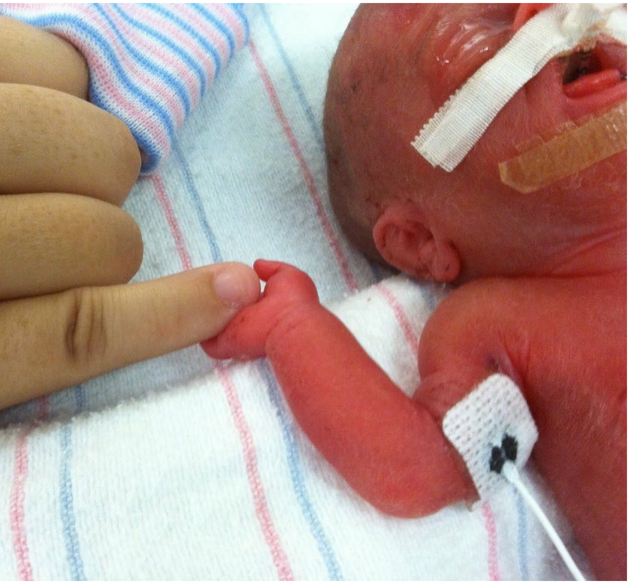

{| h.e.a.t.h.e.r |}by baby Lillian Nadine.2021

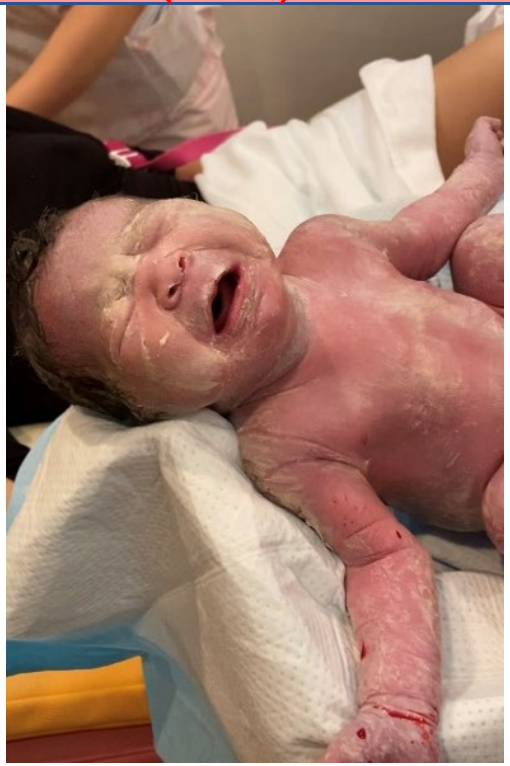

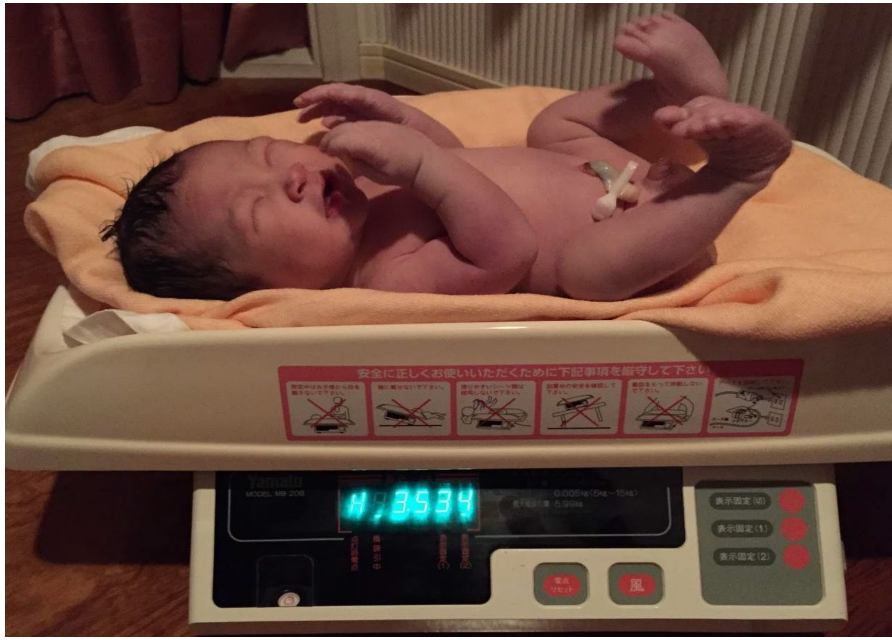

Shoko Takeuchi. 2019

About 80% of preterm babies are born late preterm

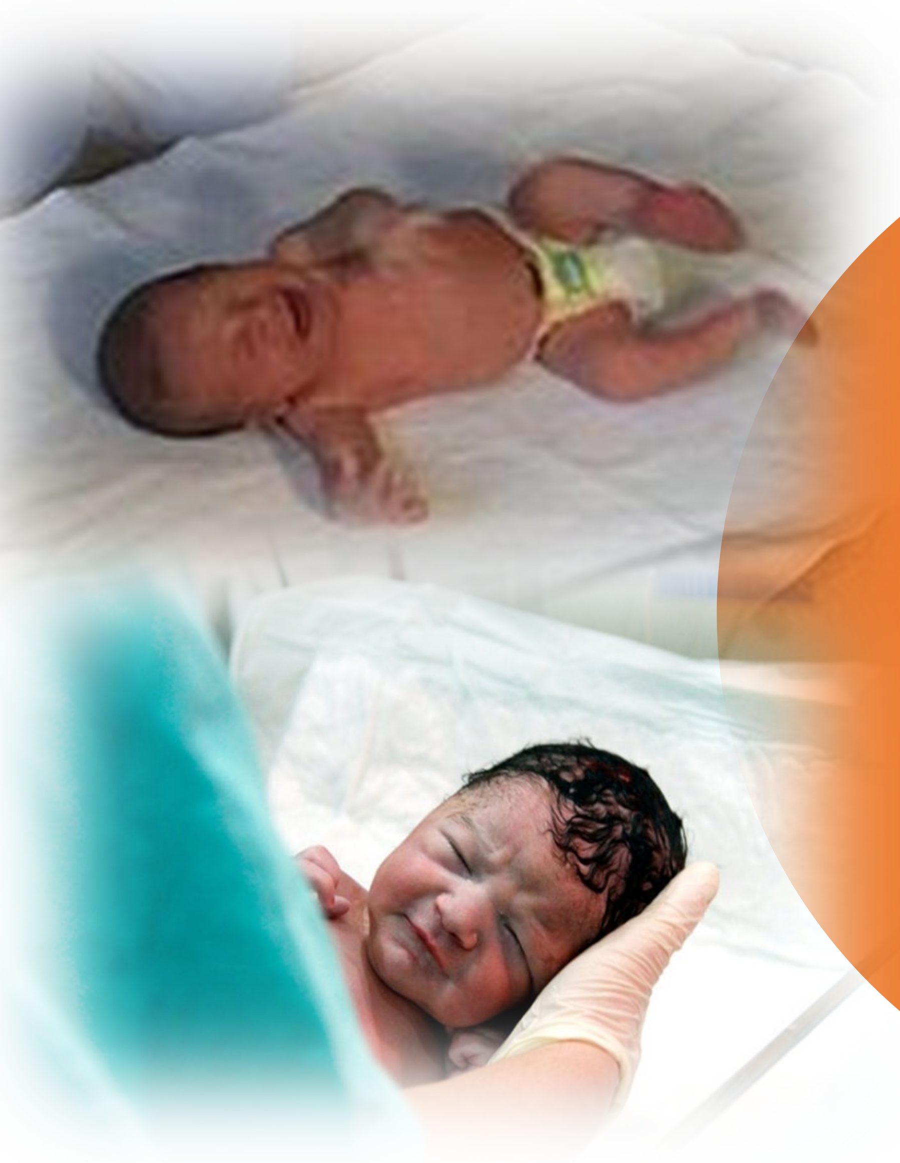

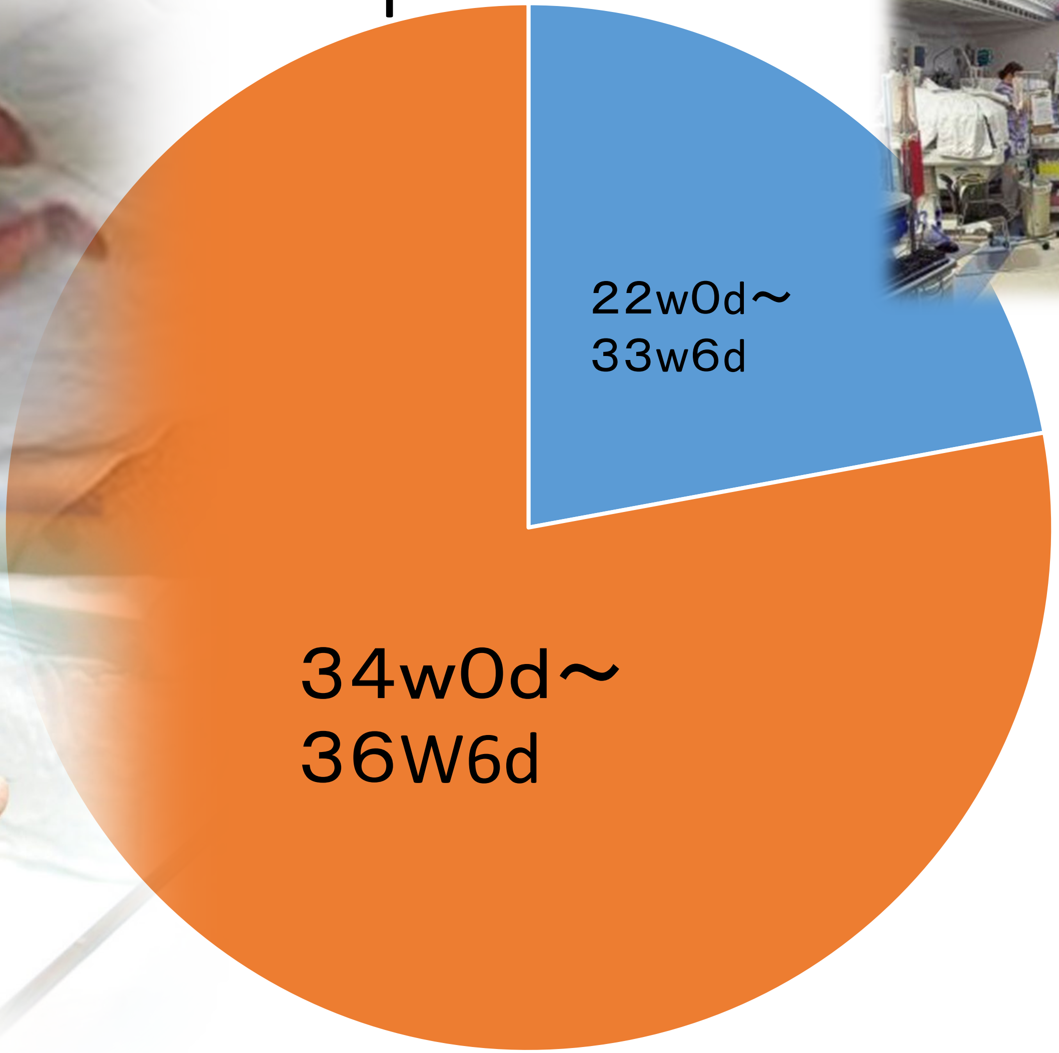

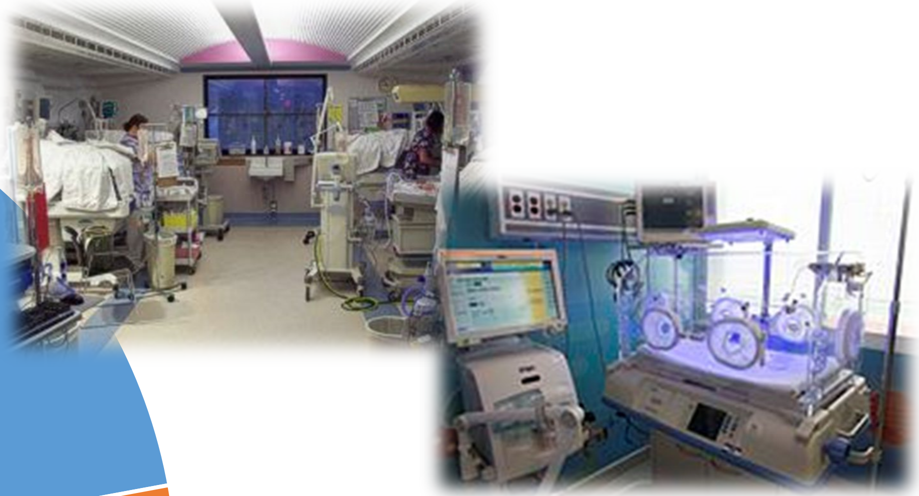

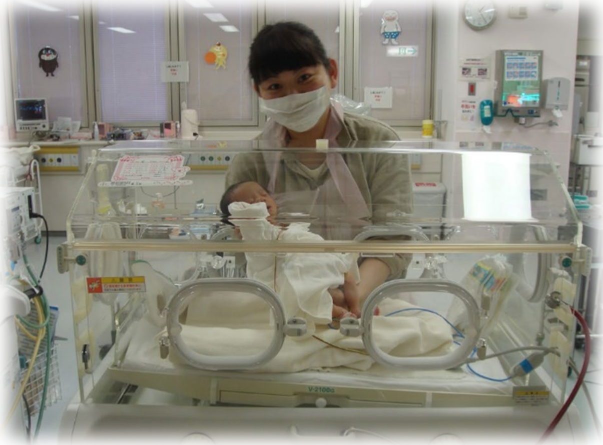

About 40% of LPIs are over 2500g

No difference in appearance from full-term infants

Managed in the maternity ward

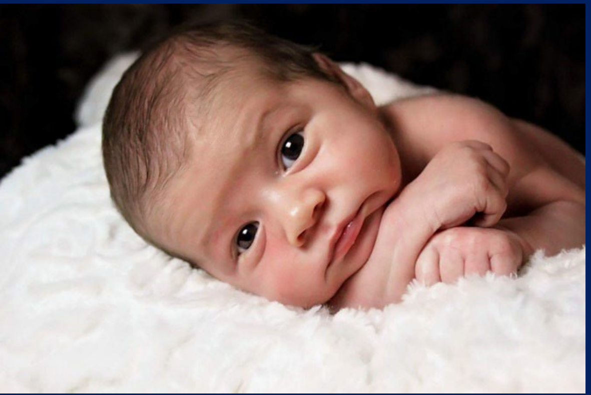

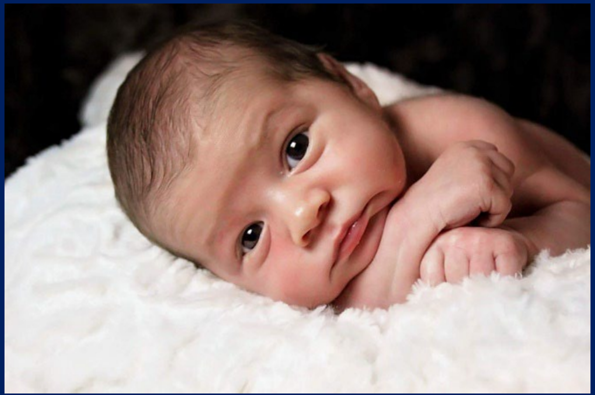

Work description

Role Assignment [1 minute].

Decide on a chairperson, a secretary, and a presenter.

Individual work (1 minute)

Read the following case study

On three sticky notes, write, "What are the characteristics of LPIs?"

Group work（4 minutes）

Separate the sticky notes into categories and paste them on the worksheet.

Case

Pregnancy week 36 weeks and 0 days, normal delivery, male, 2500 g

Water broke during the mother's hospitalization for urgent preterm labor, leading to delivery.

Baby A is in good general condition. He was managed in the maternity ward.

After birth: Day 1

Paste sticky notes in each category.

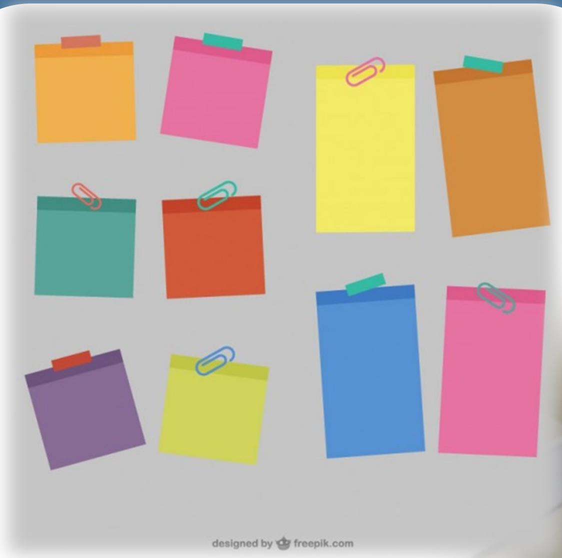

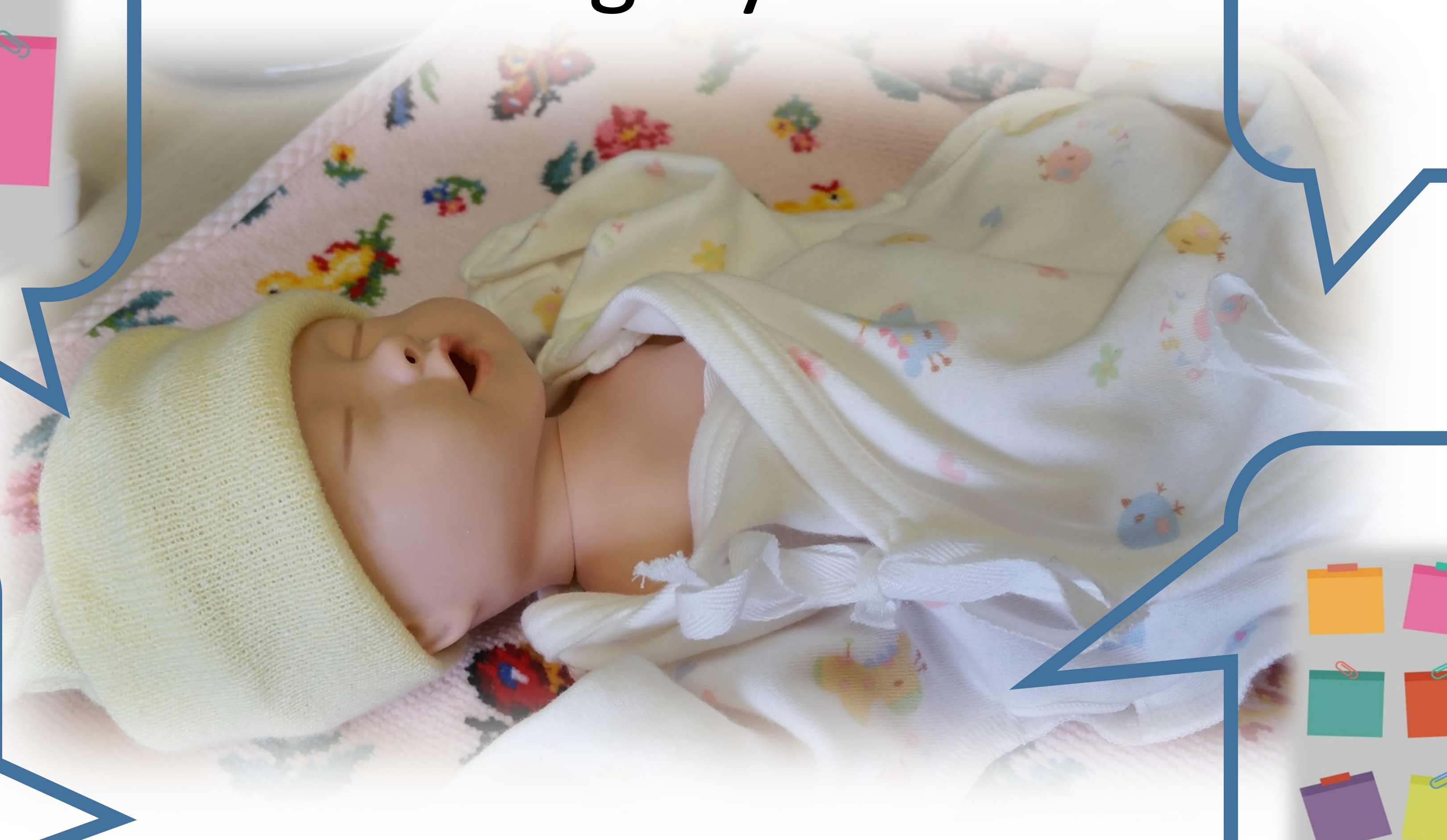

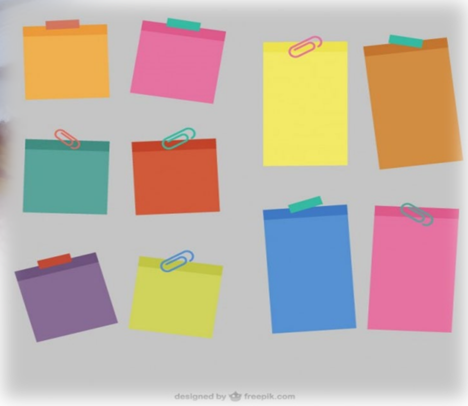

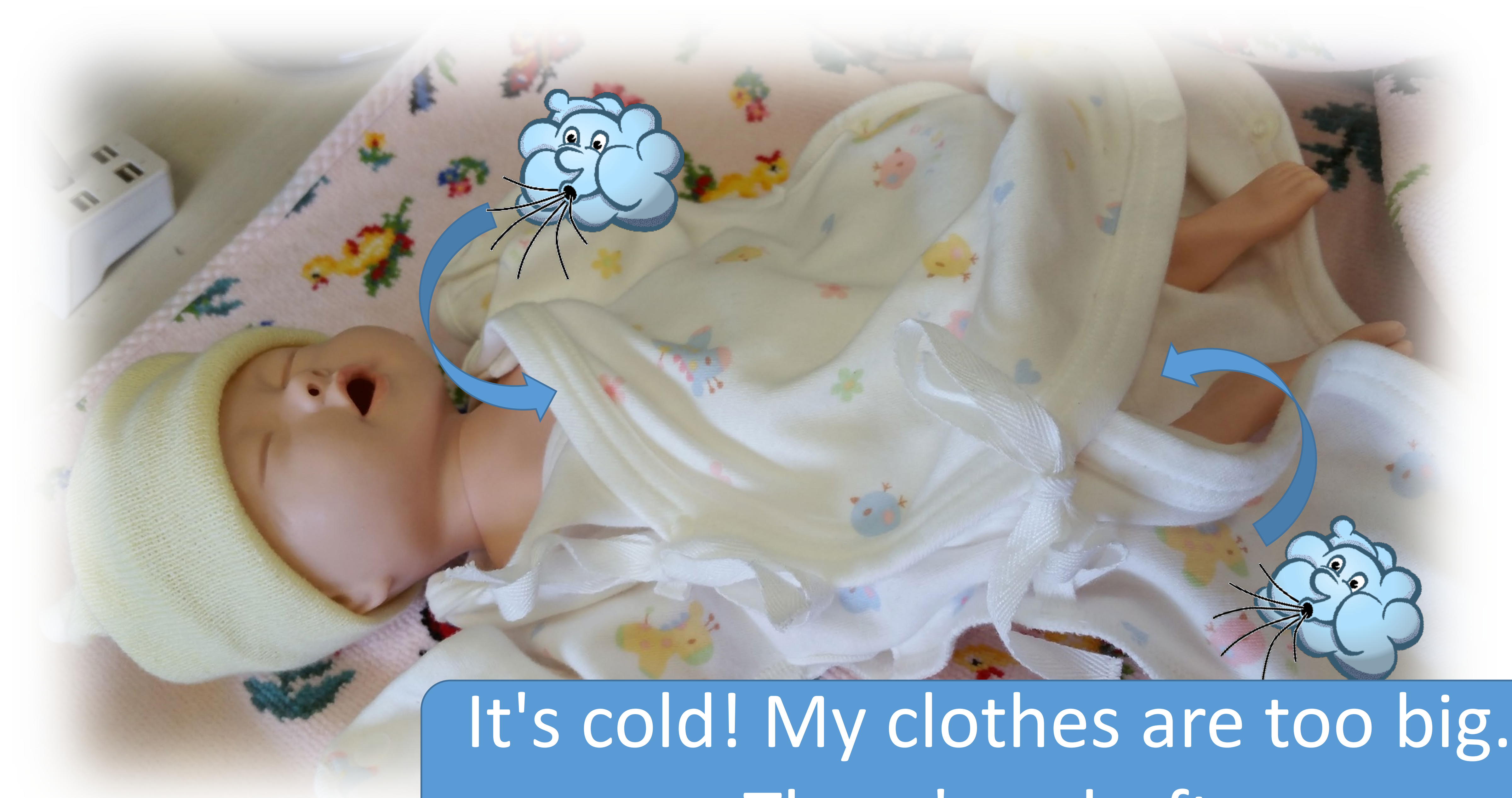

It's cold! My clothes are too big.
There's a draft.

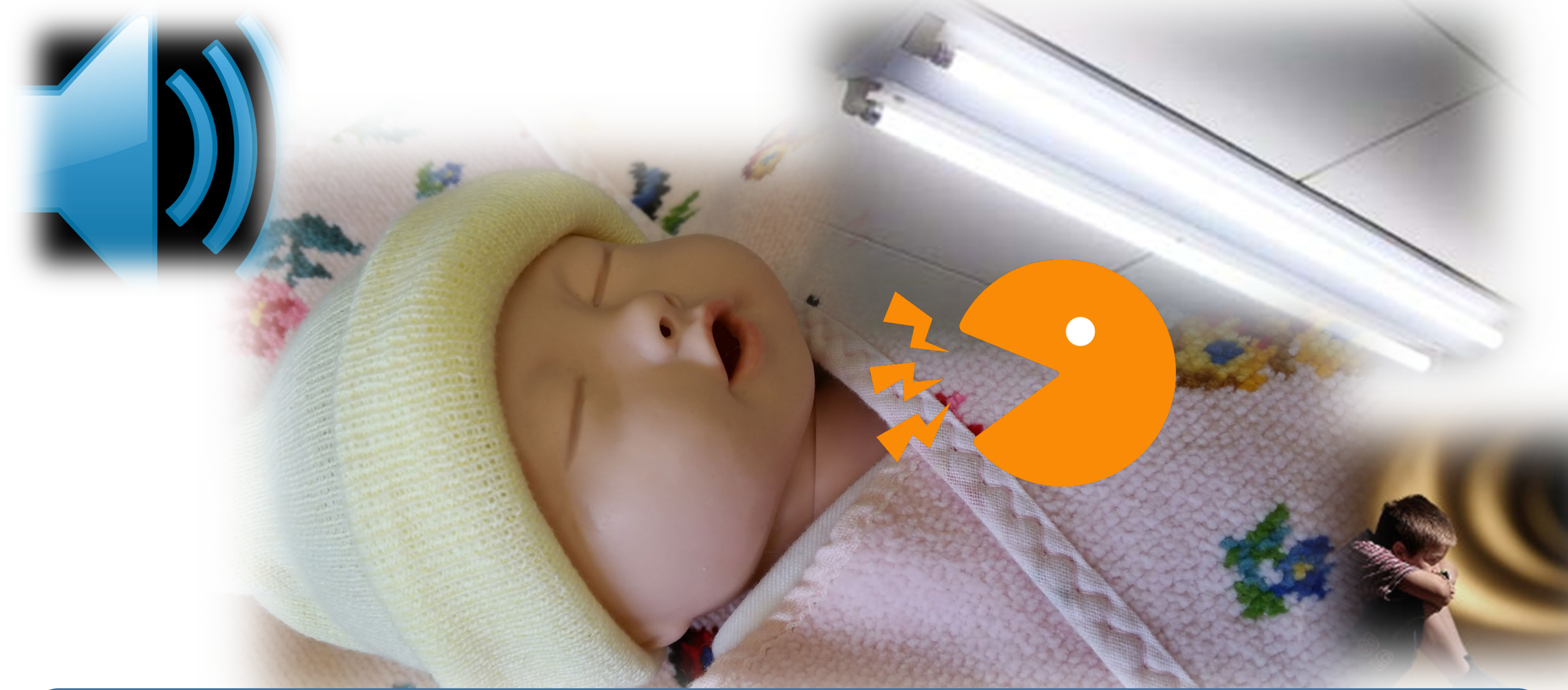

The sound of voices! Too bright! It's too dry! I need to shut myself away for a while!

Physical characteristics of LPIs

Hypothermia, hypoglycemia, excessive weight loss, dehydration

Poor weight gain, stunted growth

Prolonged supplementation of artificial milk

Increased jaundice, fever due to dehydration

Inability to establish breastfeeding

The Academy of Breastfeeding Medicine（2016）

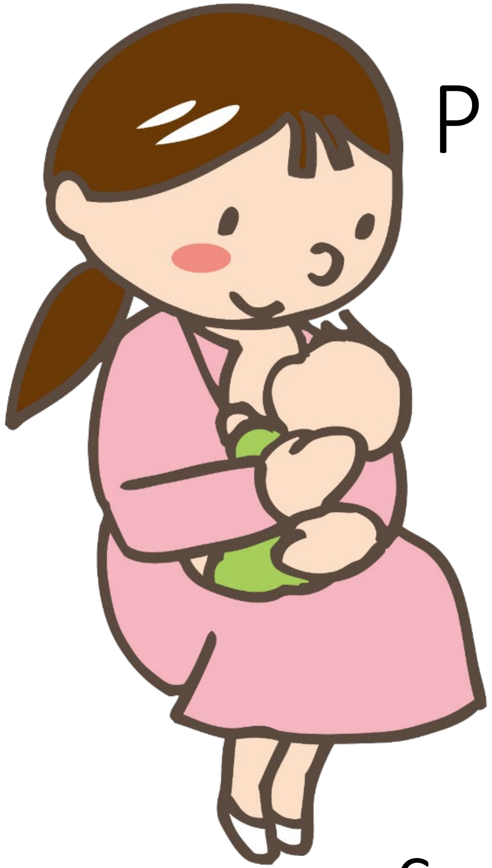

Prevention of excessive weight loss and dehydration

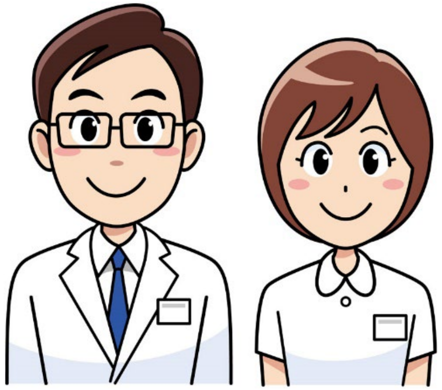

Evaluation/monitoring of breastfeeding

Is effective milk transfer occurring?

Evaluation of the amount of milk transferred

Medical treatment, breastfeeding support

・Consideration of supplementation, promotion of milk production, and maintenance

Skin-to-skin contact, etc.

（The Academy of Breastfeeding Medicine Clinical Protocol Committee #10, 2016）

IMPORTANT

- 3% of birth weight loss by 24 hours of age, and 7% loss by 72 hours, reassess breastfeeding.

Comparison of breastfed and artificially fed children

● blood sugar level

Breastfed children ≦ artificially fed children

● Ketone body concentration

Breastfed children ≧ artificially fed children

● Insulin concentration

Breastfed children ≦ Artificially fed children

IMPORTANT

Breastfeeding Late Preterm Infant activates compensatory function for hypoglycemia

IMPORTANT

In the event of breathing disorders, searching - sucking - swallowing reflexes are inhibited

Nutrients that promote neurodevelopment

- Long-chain polyunsaturated fatty acids (especially DHA)
- Insulin-like growth factor (IGF-1)
- Phospholipids (sphingomyelin)
- Taurine
- Erythropoietin

one's mother's may be included

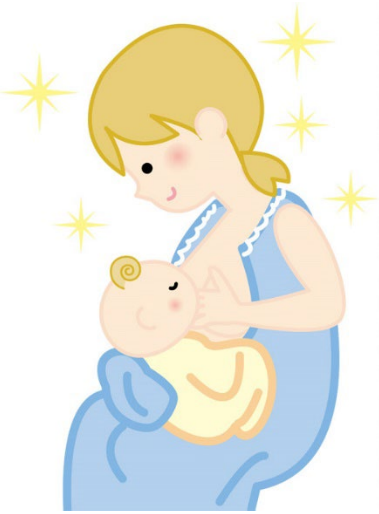

Characteristics of breastfeeding in mothers with LPIs

Breastfeeding is valuable for a preterm infant.

VS

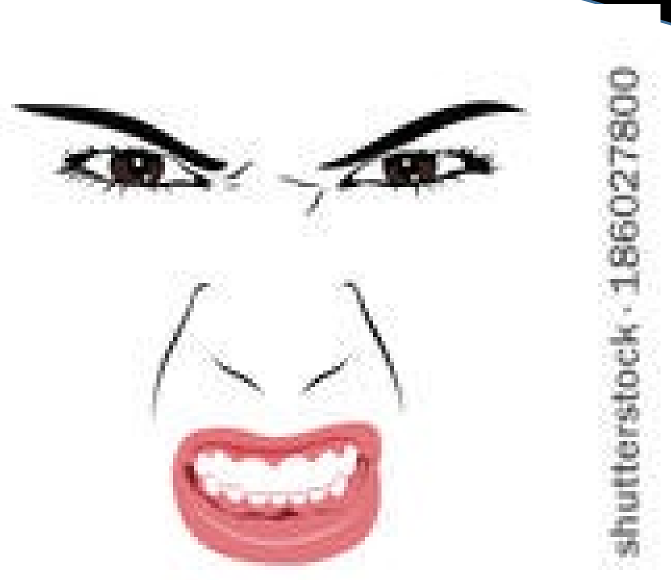

Lack of confidence in breastfeeding and feeling frustrated from feeding an LPI

Why we incorporate skin-to-skin contact to increase LPIs' ability to suckle

- To prevent decreased feeding ability due to lowered body temperature
- To increase the possibility of feeding the baby whenever the baby shows a desire to feed.
- To enable the baby to sleep on the mother's breast to recover the ability to suckle at the next feeding.
- To increase the mother's milk production.

Prerequisites for kangaroo care

Arrange for psychological and social support for the family.

Keep the family well informed and confirm their wishes for the implementation of care

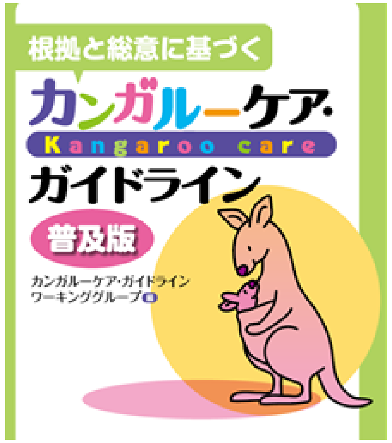

Dancer-hand position to support the jaw and cheeks during feeding = not only support but also compensate for immature functions

Increase intraoral negative pressure by applying pressure to the cheeks from both sides

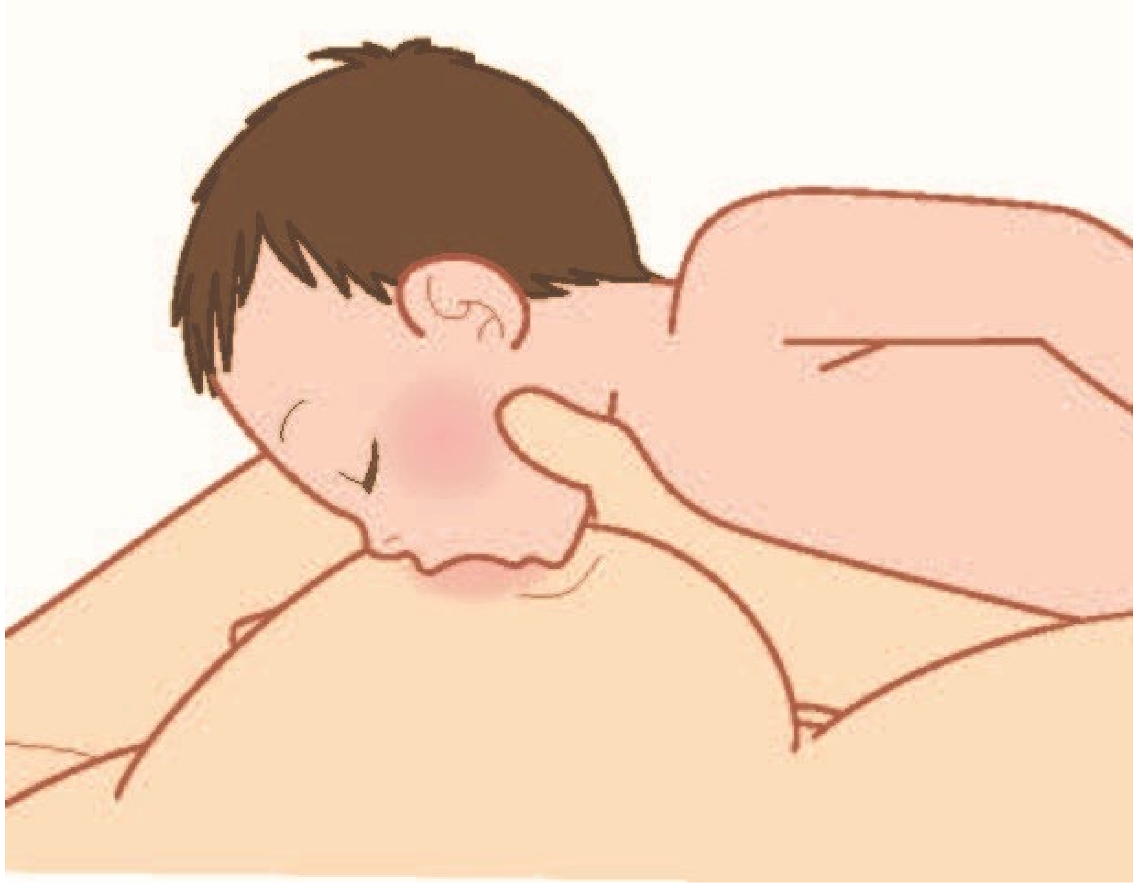

Support the chin and elevate the mandible in time with the sucking rhythm.

Pump one's own milk

When to start:

If the baby is unable to suck effectively within the first hour of life (as a temporary measure, start to feed with a spoon or dropper)

In case of mother–infant separation or low birth weight

If the baby is still sleeping or cannot suckle 24 hours after birth

The number of times you should pump:

If the baby is breastfeeding directly: 6 times/day

If the baby is not able to nurse directly at all: 8 times/day

（The Academy of Breastfeeding Medicine Clinical Protocol Committee #10, 2016）

How to solve the problem of poor weight gain (20g/day) in LPIs

- Assessing suctioning, sucking, and swallowing
- Increase frequency of feedings
- Supplement after direct feeds.
- Supplement if the child is still awake after 30–40 minutes of direct feeding. Stop direct feedings.
- Start pumping and increase frequency.
- Breastfeed after direct feeding if milk has not been fully evacuated.
- Change to a more effective milking method, such as manual or electric milking.

（The Academy of Breastfeeding Medicine Clinical Protocol Committee #10, 2016）

Work description

Role assignment（1 min）.

1st time: 1 person as nurse, 1 person as mother (except 2 people as observers)

2nd time: 1 nurse, 1 mother (all others are observers)

Individual work（2 minutes）

Read the case study

Group work（5 minutes）

Discuss information gathering, assessment, and care planning necessary for support

Simulations（6 minutes x 2 times）

Prepare for each section

When ready, start on cue

Challenges to be addressed

Today you are in charge of Ms. A. Her baby (weight at birth: 2310g) weighs 2249 g (-2.6%) on the first day of life, 2150 g (-6.9%) on the second day of life, and 2130 g (-7.7%) on the third day of life. The infant tends to sleep and may fall asleep during feedings. The mother gives him a cup of milk that she has pumped after direct feeding.

You are about to visit Ms. A's room.

What will you observe when you go to Ms. A's place? What do you observe, and what do you think needs to be done?

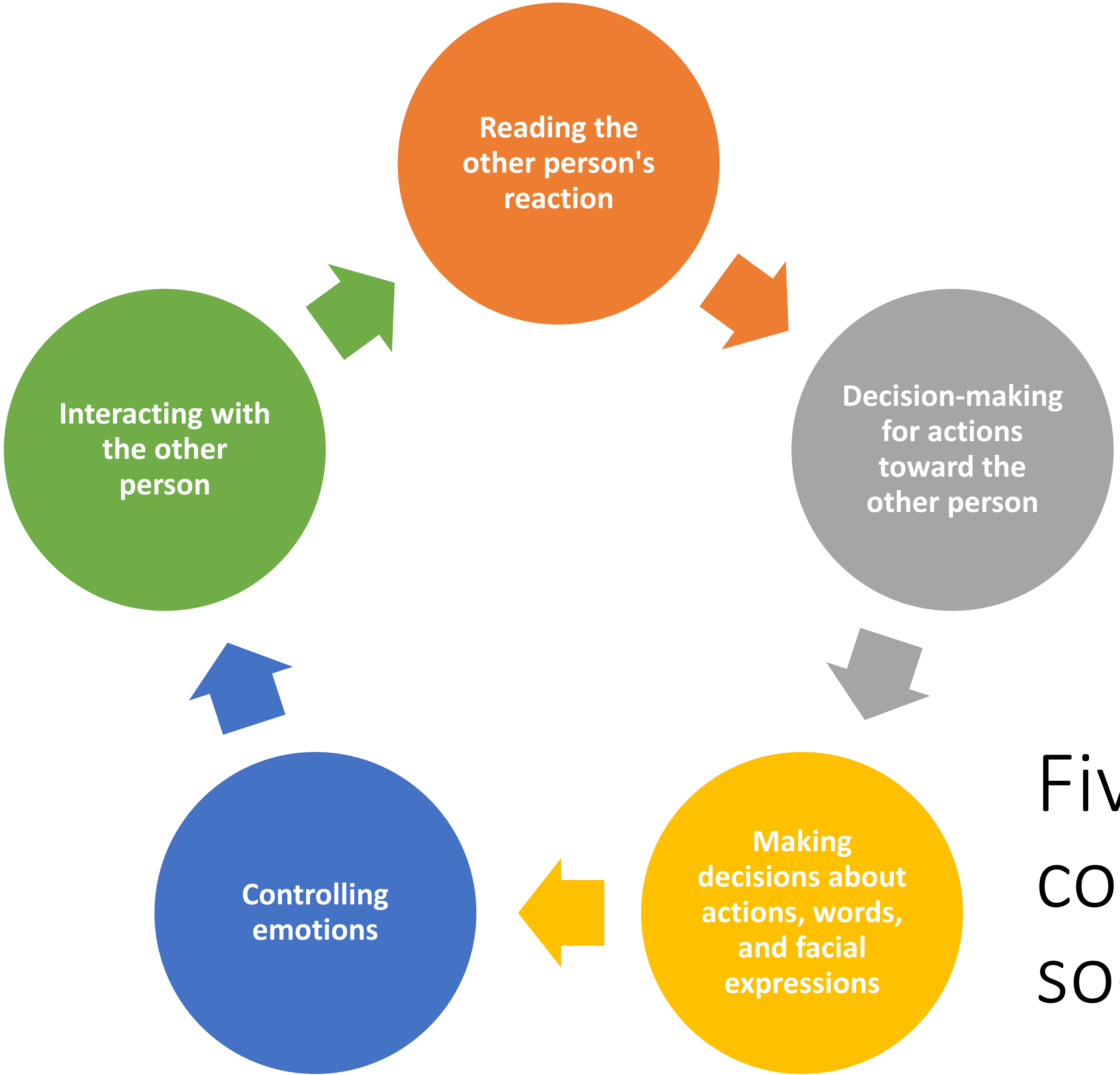

Five major components of social skills

Social skills training

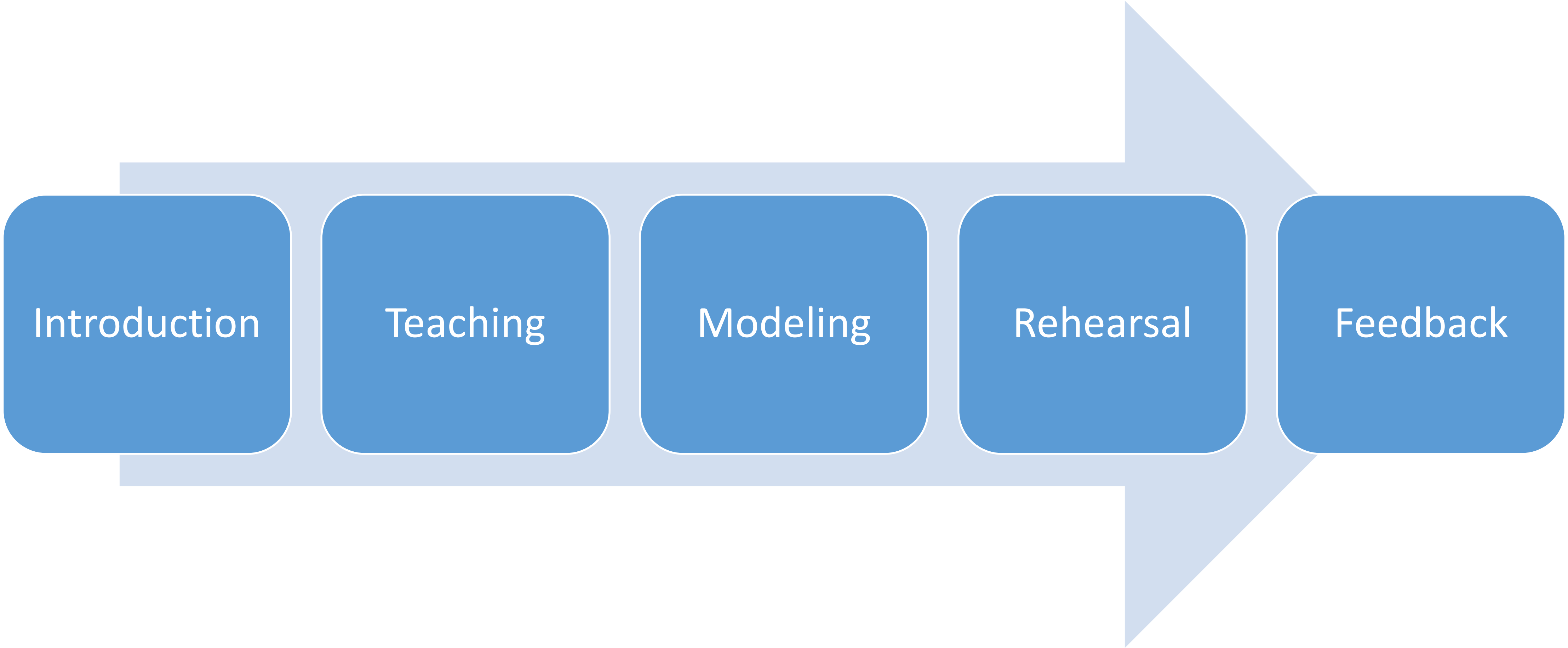

Work description

Role assignment（1 minute）: 1 person as nurse, 1 person as mother (Others are observers)

Individual work（2 minutes）: Read the case study

Group work (5 minutes): Each person in charge of the group will be responsible for their own work.

Reflection in each group（15 minutes）

The observer gives feedback on what was not done and what was done while looking at the nursing social skills sheet.

Reflection by the nurses themselves

(1) Ask the nurse to describe their feelings about the mother.

（2) How did you adjust your feelings?

（3) Thoughts on social skills

Case 3: "Breastfeeding support for mothers who refuse to pump"

On the second day of puerperium, the nurse who was in charge of Ms. A contacted me.

According to Ms. A's feeding and milking chart, she had been milking three times a day, but after midnight, she didn't seem to be milking at all.

You are the nurse in charge of Ms. A today.

Ms. A is still not milking.

When you encounter this situation, how do you respond to Ms. A?

Use the social skills you have learned to communicate with Ms. A.
