# Supplementary material for: Efficacy of a breastfeeding support education program for nurses and midwives: a randomized controlled trial
Source: Int Breastfeed J. 2022 Dec 22;17:92. doi: 10.1186/s13006-022-00532-2 (PMC9773528; doi:10.1186/s13006-022-00532-2)
Supplement: Supplementary file 4 — Additional file 4. Breastfeeding support chart for LPIs. [file 13006_2022_532_MOESM4_ESM.pdf]

Breastfeeding support chart for LPIs

During pregnancy	When planning to give birth at week 34–37 of gestation	Information provision<br>▪ Advantages of breastfeeding<br>▪ Breast milk secretion, establishment, and maintenance
During hospitalization	Immediately after birth	Assistance with continuous skin-to-skin contact<br>Initial breastfeeding: If direct breastfeeding is difficult, pump milk within one hour and place milk in baby's mouth
	Until 24 hours after birth	Breastfeeding description<br>・Benefits of breastfeeding, methods of breastfeeding and establishment, maintenance, and specific strategies<br>・ Direct breastfeeding procedure: Necessity and procedure of dancer-hand position<br>・ Breastfeeding method: As a general rule, babies should be given as much as they want. The estimated frequency of breastfeeding is 8–12 times/day (increase when weight gain is poor) and the frequency of milking is at least six times/day (eight times/day if the baby cannot suckle properly)<br>・ Timing of breastfeeding: signs of wanting to be breastfed, sleep/wake rhythm, soothing method, waking method, etc.<br>・ Milking: Explanation of the necessity, method, and items used for milking<br>Environmental adjustment<br>・ Mother and child sharing room all day: Providing a place where the mother can stay near the child if not possible<br>・ Continuous skin-to-skin contact: Performed without restrictions unless there is a valid reason<br>Prevention of rapid weight loss<br>・ If the weight loss rate is 3% or more at 24 hours after birth, re-evaluation of breastfeeding
	Until 72 hours	Prevention of rapid weight loss

	after birth	・ If the weight loss rate is 7% or more at 24 hours after birth, re-evaluation of breastfeeding method<br>Prevention of insufficient feeding based on direct breastfeeding<br>・ When necessary, select an auxiliary tool that facilitates the transition to direct breastfeeding. First, use a cup or spoon. Use artificial nipples only for good reason.<br>・ Do not reduce the frequency of direct breastfeeding by pouring milk from the corner of the mouth using a tube while breastfeeding directly.
	Until the day before discharge	Evaluation for discharge<br>・ Determine whether the child is physiologically stable and able to take in a sufficient amount of milk with only or supplemented breast milk.<br>Post-discharge breastfeeding plan<br>▪ Determining situation-specific breastfeeding method that prioritizes direct breastfeeding<br>▪ Procuring breastfeeding support equipment and confirming actual use<br>▪ Trying manual breast pump and electric breast pump and preparing for continuous use even after discharge when necessary<br>▪ Documenting post-discharge breastfeeding plan and sharing it with the mother and post-discharge breastfeeding specialists.<br>▪ Confirming contact method with the decision of a breastfeeding support specialist outside the delivery facility<br>Date of first consultation after discharge<br>▪ Confirm the first consultation appointment after discharge within 48 hours after discharge<br>▪ Setup to ensure that mothers and children can receive medical examinations<br>Explanation to family and request for cooperation
After discharge	Until the second day	Observation content during first consultation after discharge<br>▪ Evaluate and revise status of spending two days at home based on the discharge plan

	after discharge	・ Provide lifestyle advice to continue breastfeeding<br>Confirmation of next consultation appointment<br>・ If there are changes in the breastfeeding plan, the consultation will be done within 48 hours. Otherwise, regular follow-up will be conducted once a week.
	Until 40 weeks after conceptions	Period of regular follow-up<br>・ Weekly weight check until 40 weeks after conception or until proven that the LPI is gaining weight without supplementation<br>Timing of follow-up in addition to regular follow-up<br>・ Consultation when weight gain is poor<br>・ 2–4 days after changing the breastfeeding plan<br>Follow-up content<br>・ Confirm of weight gain and determining poor weight gain (less than 20g/day)<br>・ Check height and head circumference, average 0.5 cm/week<br>・ Bilirubin check when needed<br>・ Introduce breastfeeding support specialists<br>・ Evaluate children who have difficulty suckling<br>・ Consider drug use in case of insufficient milk production<br>・ Evaluate whether the mother is following the breastfeeding plan and change the plan when necessary<br>・ Prevent anemia in children
