# Supplementary material for: Efficacy of a breastfeeding support education program for nurses and midwives: a randomized controlled trial
Source: Int Breastfeed J. 2022 Dec 22;17:92. doi: 10.1186/s13006-022-00532-2 (PMC9773528; doi:10.1186/s13006-022-00532-2)
Supplement: Supplementary file 5 — Additional file 5. Knowledge and skills necessary for breastfeeding support for LPIs (K-S test). [file 13006_2022_532_MOESM5_ESM.pdf]

Knowledge and skills necessary for breastfeeding support for LPIs (K-S test)

I. The following questions are related to breastfeeding support for mothers of late preterm infants (LPIs).

１． Which of the following statements about LPIs is correct?

(1) LPIs are newborns born between 35 weeks 0 days and 36 weeks 6 days of age.
(2) LPIs are treated in the same way as normal newborns when the birth weight is 2,500 g or more and the infant is not a "low birth weight infant.
(3) In Japan, LPIs account for about 80% of all preterm births.
(4) LPIs are appropriately referred to as "near-term" in the sense that they are newborns who are close to full-term infants among preterm infants.

２． Which of the following is not true about breastfeeding LPIs?

(1) LPIs are best nursed in the sleep-wake state (State) 3 to 5.
(2) LPIs may increase their milk intake through sustained skin-to-skin contact with their mothers.
(3) LPIs' suckling is not affected by respiratory status, and exploration-sucking-swallowing is reflexive.
(4) LPIs may have difficulty gaining weight even if they consume milk as well as full-term infants.

３． Which of the following is the correct method for the first feeding of LPIs?

(1) Use a bottle.
(2) Use a syringe.
(3) Use a cup.
(4) Direct breastfeeding.

４． Which statement about breastfeeding support for mothers who have given birth to LPIs is correct?

(1) Since the delivery of LPIs was scheduled, a plan was made so that after the delivery,

the mother would be given an explanation of the benefits of breastfeeding and how to breastfeed after confirming that the child was in good health.
(2) The time for teaching breastfeeding should be after the increase in milk production is confirmed.
3) Early mother-infant contact after delivery reduces the incidence of LPIs.
(4) Breastfeeding of LPIs requires active milking, which should be done every 2 hours immediately after delivery and at night.

５． Which statement about prevention of hypothermia in LPIs is incorrect?

(1) Because LPIs lose a lot of heat from the body surface, they should wear clothing during skin-to-skin contact.
(2) To prevent LPIs from losing body heat, it is recommended that they wear more clothing and a hat.
(3) LPIs should not be bathed until their body temperature has stabilized.
(4) LPIs should have their temperature taken at two different points.

６． Which statement is false about LPIs that do not attempt to feed?

(1) Initiate feeding during shallow wakefulness or when the baby is not too hungry.
(2) If the child is sleeping, take a blanket, talk to him, and massage his hands and feet to wake him up.
3) Drape the milk around the baby's mouth.
(4) Give milk by bottle instead of direct feeding so as not to exhaust the child.

７. Which statement about jaundice in LPIs is incorrect?

1) Encourage frequent feedings from early in life.
(2) Administer water or sugar water to LPIs to increase defecation and reduce jaundice.
(3) Explain to mothers that the excretion of their feces reduces the enterohepatic circulation of bilirubin and prevents jaundice and encourage active breastfeeding.
(4) Prevent jaundice by increasing caloric intake through sugar water and other supplements.

８. Which statement about hypoglycemia in LPIs is correct?

(1) All LPIs should be subject to routine glucose screening.
(2) Breast-fed LPIs were given artificial milk to prevent hypoglycemia because of their active desire to suckle and intense crying.
(3) Low blood glucose levels in breastfed infants promote ketone body production, which can be used as an alternative energy source to glucose.
(4) LPIs born to mothers who used ritodrine during pregnancy are more likely to develop hyperglycemia.

９． Which statement about brain development in LPIs is incorrect?

(1) The brain weight of LPIs is about 65% to 75% of that of full-term infants.
(2) It takes 4 to 6 weeks for the brain weight of LPIs to reach that of full-term infants.
3) The neural network matures rapidly between 30 and 34 weeks of gestation.
(4) Interruption of brain development due to shortened gestational weeks may pose a risk of behavioral and mental problems throughout life.

１０． Which statement about support for LPIs is incorrect?

(1) Preventing hypoglycemia and jaundice to prevent invasion of the immature brains of LPIs.
(2) Encourage mothers to breastfeed so that LPIs can obtain polyunsaturated fatty acids and cholesterol after birth through breast milk, which they were unable to obtain in utero.
(3) Increase the duration of wakefulness by actively providing sound and light stimulation to promote the neurological development of LPIs.
(4) Check whether mothers with LPIs feel any inconvenience in the environment for raising their children and encourage them to utilize social resources when necessary.

II. Please provide the appropriate number for (A) through (J).

１． To prevent excessive weight loss in LPIs, lactation should be reevaluated if weight loss from birth weight exceeds (A) percent by 24 hours of age or (B) percent by 72 hours of age.

２． LPIs should have a minimum weight gain of (C) g/day and height and head growth of (D) cm/week each until 40 weeks after conception or until growth is steady. 3.

３. LPIs should be encouraged to have their first feeding within (E) hours of birth, and in the case of mother-infant separation, mothers should begin hand pumping within (F) hours of birth. If the baby cannot be suckled within (G) hours of birth, start milking by hand or with a milking device.

４． It is recommended that kangaroo mothers care for low birth weight infants with good general health should be done for at least (H) minutes per session.

５． Supplementation should be done if the infant is still awake and unsatisfied after a maximum of (I) minutes of direct feeding of LPIs. 6.

６． The dancer-hand position is a feeding method in which the mother supports the breast and the child's lower jaw with (J) fingers.
