# Supplementary material for: Efficacy of a breastfeeding support education program for nurses and midwives: a randomized controlled trial
Source: Int Breastfeed J. 2022 Dec 22;17:92. doi: 10.1186/s13006-022-00532-2 (PMC9773528; doi:10.1186/s13006-022-00532-2)
Supplement: Supplementary file 6 — Additional file 6. Scale development: knowledge and skills necessary for breastfeeding support for LPIs. [file 13006_2022_532_MOESM6_ESM.pdf]

Scale Development: Knowledge and skills necessary for breastfeeding support for LPIs

We created a 20-item questionnaire evaluating knowledge and skills (K-S) to assess participants' knowledge of breastfeeding support for LPIs. It consisted of two items on basic knowledge about LPIs, 14 items on knowledge and skills related to breastfeeding support for their mothers, and four items on knowledge of systemic management of LPIs (additional file 5: The Knowledge and Skills (K-S test)). The items scored five points for each correct answer, and the total score ranged from 0 to 100. Higher scores indicated better knowledge and skills.

The same test was performed at regular intervals to evaluate the consistency of the measurement methods. A regression equation was created in a Bland–Altman plot with the difference between the two measurements on the y-axis and the average of the two measurements on the x-axis. The average difference between the measured values was close to 0, and both positive and negative values were distributed almost evenly within the permissible range of error (Figure 1). Since the 95% confidence interval of the difference between the first and second measurements of the addition error was -5.7 to 6.2, $p = 0.92$, and the significance of the regression of the proportional error was $p = 0.30$, no systematic error was observed in either case. Thus, it was confirmed that the same measurement items were measured separately for the first and second times and that the measurement methods were consistent.

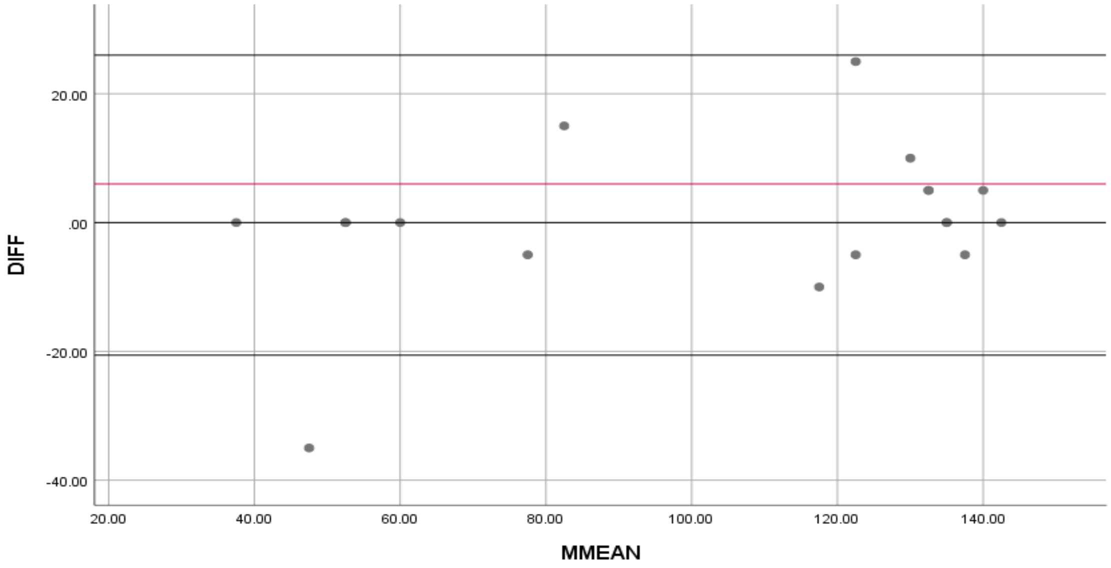

Figure 1: Bland–Altman plot
