# Supplementary material for: Efficacy of a breastfeeding support education program for nurses and midwives: a randomized controlled trial
Source: Int Breastfeed J. 2022 Dec 22;17:92. doi: 10.1186/s13006-022-00532-2 (PMC9773528; doi:10.1186/s13006-022-00532-2)
Supplement: Supplementary file 7 — Additional file 7. Cochrane review risk of bias checklist. [file 13006_2022_532_MOESM7_ESM.pdf]

Cochrane Review Risk of Bias Checklist

Item to Check	Result
Appropriate allocation procedure	Allocation was performed using stratified randomization table software to automatically allocate participants to two groups and four blocks.
Allocation concealment	Study participants were assigned a number when allocation was performed using the allocation table. Therefore, the method adopted did not allow identification of individuals or for researchers to guess which group participants were assigned to.
Blinding	This was a single-blind study. Only research participants were blinded.
Incomplete outcome data	The ITT principle was adhered to in cases that could not be followed up.
Selective outcome reporting	Outcomes were explained in the research method.
Other bias	There were no instances of early termination of the study.
