# Supplementary material for: Efficacy of a breastfeeding support education program for nurses and midwives: a randomized controlled trial
Source: Int Breastfeed J. 2022 Dec 22;17:92. doi: 10.1186/s13006-022-00532-2 (PMC9773528; doi:10.1186/s13006-022-00532-2)
Supplement: Supplementary file 8 — Additional file 8. CONSORT 2010 checklist. [file 13006_2022_532_MOESM8_ESM.pdf]

Table 1 | CONSORT 2010 checklist of information to include when reporting a randomized trial* Izumi SATO

**Section/Topic**	**Item No**		**Checklist item Reported on line**
**Title and abstract**			
	1a	Identification as a randomized trial in the title	**Line: 1**
	1b	Structured summary of trial design, methods, results, and conclusions (for specific guidance see CONSORT for abstracts 45 65)	**Lines: 17–46**
**Introduction**			
Background and objectives	2a	Scientific background and explanation of rationale	**Lines: 93–106**
	2b	Specific objectives or hypotheses	**Lines: 107–110**
**Methods**			
Trial design	3a	Description of trial design (such as parallel, factorial) including allocation ratio	**Lines: 115–118**
	3b	Important changes to methods after trial commencement (such as eligibility criteria), with reasons	
Participants	4a	Eligibility criteria for participants	**Lines: 131–137**
	4b	Settings and locations where the data were collected	**Lines: 274–280**
Interventions	5	The interventions for each group with sufficient details to allow replication, including how and when they were actually administered	**Lines: 176–184**
Outcomes	6a	Completely defined pre-specified primary and secondary outcome measures, including how and when they were assessed	**Lines: 287–315**
	6b	Any changes to trial outcomes after the trial commenced, with reasons	
Sample size	7a	How the sample size was determined	**Lines: 162–174**
	7b	When applicable, explanation of any interim analyses and stopping guidelines	
Randomization:			
Sequence generation	8a	Method used to generate the random allocation sequence	**Lines: 138–142**
	8b	Type of randomization; details of any restriction (such as blocking and block size)	**Lines:138~142**
Allocation concealment mechanism	9	Mechanism used to implement the random allocation sequence (such as sequentially numbered containers), describing any steps taken to conceal the sequence until interventions were assigned	**Lines: 143–147**
Implementation	10	Who generated the random allocation sequence, who enrolled	**Lines: 148–161**

		participants, and who assigned participants to interventions	
Blinding	11a	If done, who was blinded after assignment to interventions (e.g., participants, care providers, those assessing outcomes) and how	**Lines: 148–161**: Participants were blinded only to the name of the program, venue, date, and time of their participation, and not to whether they were assigned to the intervention or control group.<br>However, the intervention was administered by the researchers themselves.<br>The research collaborators carefully checked the interventionists' faithful implementation of the program scenario during the program implementation. Post-intervention evaluation measures were tabulated by the research collaborators. The researcher received the aggregated data. This prevented the falsification of the data by the researchers.<br>The researcher did not make any minor adjustments to the data after tabulation and conducted the analysis based on the research plan.<br>The intervention was carried out by the researchers themselves. The method of blinding may not be sufficient.
	11b	If relevant, description of the similarity of interventions	
Statistical methods	12a	Statistical methods used to compare groups for primary and secondary outcomes	**Lines: 281–315**
	12b	Methods for additional analyses, such as subgroup analyses and adjusted analyses	**Lines: 311–315**
**Results**			
Participant flow (a diagram is strongly recommended)	13a	For each group, the numbers of participants who were randomly assigned, received intended treatment, and were analyzed for the primary outcome	**Lines: 321–332**
	13b	For each group, losses, and exclusions after randomization, together with reasons	**Lines: 322–332**
Recruitment	14a	Dates defining the periods of recruitment and follow-up	**Lines: 275–276**
	14b	Why the trial ended or was stopped	
Baseline data	15	A table showing baseline demographic and clinical characteristics for each group	Table 4
Numbers analyzed	16	For each group, the number of participants (denominator) included in each analysis and whether the analysis was conducted by original assigned groups	**Lines: 326–332**
Outcomes and estimation	17a	For each primary and secondary outcome, results for each	**Lines: 314–315**

		group, and the estimated effect size and its precision (such as 95% confidence interval)	
	17b	For binary outcomes, presentation of both absolute and relative effect sizes is recommended	
Ancillary analyses	18	Results of any other analyses performed, including subgroup analyses and adjusted analyses, distinguishing pre-specified from exploratory	**Lines: 383–426**
Harms	19	All important harms or unintended effects in each group (for specific guidance see CONSORT for harms42)	
**Discussion**			
Limitations	20	Trial limitations, addressing sources of potential bias, imprecision, and, if relevant, multiplicity of analyses	**Lines: 495–549**
Generalizability	21	Generalizability (external validity, applicability) of the trial findings	**Lines: 472–494**
Interpretation	22	Interpretation consistent with results, balancing benefits and harms, and considering other relevant evidence	**Lines: 430–471**
**Other information**			
Registration	23	Registration number and name of trial registry	**Line 43:** The study was retrospectively registered (UMIN, UMIN000035227) on December 12, 2018.
Protocol	24	Where the full trial protocol can be accessed, if available	
Funding	25	Sources of funding and other support (such as supply of drugs), role of funders	**Lines: 576–578**
